# RASP: Optimal Single Puncta Detection in Complex Cellular Backgrounds

**DOI:** 10.1021/acs.jpcb.4c00174

**Published:** 2024-04-09

**Authors:** Bin Fu, Emma E. Brock, Rebecca Andrews, Jonathan C. Breiter, Ru Tian, Christina E. Toomey, Joanne Lachica, Tammaryn Lashley, Mina Ryten, Nicholas W. Wood, Michele Vendruscolo, Sonia Gandhi, Lucien E. Weiss, Joseph S. Beckwith, Steven F. Lee

**Affiliations:** † Yusuf Hamied Department of Chemistry, University of Cambridge, Lensfield Road, Cambridge CB2 1EW, U.K.; ‡ Aligning Science Across Parkinson’s (ASAP) Collaborative Research Network, Chevy Chase, Maryland 20815, United States; § Centre for Misfolding Diseases, Yusuf Hamied Department of Chemistry, University of Cambridge, Cambridge CB2 1EW, U.K.; ∥ The Queen Square Brain Bank for Neurological Disorders, Department of Clinical and Movement Neuroscience, UCL Queen Square Institute of Neurology, London WC1N 3BG, U.K.; ⊥ Department of Neurodegenerative Diseases, UCL Queen Square Institute of Neurology, London WC1N 3BG, U.K.; # Department of Clinical and Movement Neurosciences, UCL Queen Square Institute of Neurology, London WC1N 3BG, U.K.; ¶ The Francis Crick Institute, King’s Cross, London NW1 1AT, U.K.; ∇ Great Ormond Street Institute of Child Health, University College London, London WC1E 6BT, U.K.; ○ UK Dementia Research Institute at the University of Cambridge, Cambridge CB2 0AH, U.K.; ⧫ Department of Clinical Neurosciences, School of Clinical Medicine, University of Cambridge, Cambridge CB2 0SP, U.K.; †† Department of Engineering Physics, Polytechnique Montréal, Montréal, Québec H3T 1J4, Canada

## Abstract

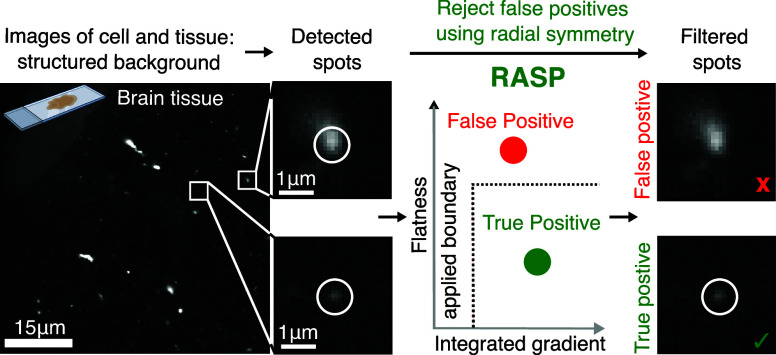

Super-resolution and single-molecule microscopies have been increasingly applied to complex biological systems. A major challenge of these approaches is that fluorescent puncta must be detected in the low signal, high noise, heterogeneous background environments of cells and tissue. We present RASP, Radiality Analysis of Single Puncta, a bioimaging-segmentation method that solves this problem. RASP removes false-positive puncta that other analysis methods detect and detects features over a broad range of spatial scales: from single proteins to complex cell phenotypes. RASP outperforms the state-of-the-art methods in precision and speed using image gradients to separate Gaussian-shaped objects from the background. We demonstrate RASP’s power by showing that it can extract spatial correlations between microglia, neurons, and α-synuclein oligomers in the human brain. This sensitive, computationally efficient approach enables fluorescent puncta and cellular features to be distinguished in cellular and tissue environments, with sensitivity down to the level of the single protein. Python and MATLAB codes, enabling users to perform this RASP analysis on their own data, are provided as Supporting Information and links to third-party repositories.

## 1 Introduction

Developments in super-resolution and single-molecule fluorescence microscopy methods continue to push the boundaries of what researchers can observe in complex biological systems. Recent examples include Moon et al., who used a combined super-resolution and spectral imaging approach to uncover the heterogeneity of live mammalian cells with ∼30 nm spatial resolution, finding chemical polarity differences in organelle and cellular membranes due to differing cholesterol levels.^1^ Deguchi et al. were able to observe single 8 nm substeps of the motor protein kinesin-1 as it “walked” on microtubules in living cells using the super-resolution technique MINFLUX.^2^ More recently, Reinhardt et al. have used a DNA barcoding method to push the spatial resolution of super-resolution method to the Ångström level for biomolecules in whole intact cells, as well as to resolve the distance between single bases in the DNA backbone.^3^ This begins to close the gap between the length scales of super-resolution microscopy and structural biology, opening up the possibility that precise structural understanding could be brought to live cells and complex tissues. All of these methods, at their core, rely on the detection of single fluorescent spots, or *puncta*. Much effort has thus been put into detecting single fluorescent puncta even when such a signal is extremely weak.

As well as identification of single fluorescent puncta, it is advantageous to simultaneously detect the large-scale surrounding cellular context, for example, in complex tissues. This enables researchers to both interrogate single molecules, such as proteins, DNAs, or RNAs, as well as understand their interaction and localization within their environments. Single-molecule fluorescence in situ hybridization (smFISH), a technique that enables the visualization of RNAs in their real biological environments, is in essence based on this principle—RNAs are detected as single bright fluorescent puncta, and the cellular or subcellular environment is imaged concurrently.^4^ smFISH has hugely improved our understanding of RNA localization and tracking and is one of the suite of techniques relied on by large-scale mapping programs such as the Allen Brain Atlas project.^5^ To give but a few examples, Shaffer et al. showed that human melanoma cells can display transcriptional variability at the single-cell level using smFISH and that this variability was a predictor of which cells would resist drug treatments in cancer.^6^ Weidemann et al. were able to use smFISH to show that the stochastic variation of gene expression was less than what might be expected from simple statistical arguments, suggesting that eukaryotes have optimized gene expression to ensure reliable cellular functions.^7^ Zhang et al. have created a spatially resolved “cell atlas” of the mouse primary motor cortex (300,000 cells) using a smFISH-based technology.^8^ More recently, Zhao et al. have shown that by combining smFISH and the use of fluorescent reporter proteins, they could quantify RNA and proteins in whole plants with subcellular resolution.^9^

There exists an underlying challenge in all of these classes of experiments: the accurate detection of and compensation for the background. In most conventional single-molecule and super-resolution experiments, sample choice and/or preparation typically is chosen to minimize the unwanted background signal. Background in this context is the combination of unwanted photons, whether from emitters or scatterers, and/or camera readout noise not related to the target molecules/process of interest. In experiments where the only photons should be from the single molecules of interest, the signal-to-background ratio can be on the order of 3–10 or more.^12^ Importantly, such an experiment’s background level would be effectively homogeneous, arising from dark counts on the detector and scattering from the solvent, in the best case.^12^ Thus, any analysis on images taken in such a single-molecule experiment is conceptually simple: bright fluorescent puncta arise from a single fluorophore on top of a homogeneous background. Such an approach has had great success in the single-molecule literature, being a frequent key step in data analysis.^13^ In more complex samples such as cellular and tissue samples (packed with intra- and/or extracellular constituents), a large variety of molecules and structures can also *autofluoresce*, i.e., emit light after excitation with the same laser used to excite a fluorescently labeled sample—this was shown elegantly by Aubin^14^ and exploited as a means to image cellular processes by König et al.,^15^ among many others.^16^ It is this spatially variant autofluorescence that causes a (conventional) simple thresholding approach to fail. The reason it fails is that the autofluorescence is related to the concentration of the water, proteins, lipids, and nucleic acids that, among other things, make up the intra- and extracellular components. These molecules are not heterogeneously distributed spatially, and thus, different areas of the cells and tissue slices will autofluoresce in a highly heterogeneous way.^17^ This creates to what we will herein refer to as structured background, after Möckl et al.,^18^ in the images of interest.

The effect of this structured background compounds the difficulty of performing single-molecule microscopy in cell specimens and tissue samples because a new approach to spot identification is needed. Hoogendoorn et al. studied this and found that structured background can cause sufficiently large artifacts in super-resolution microscopy that they defeat the purpose of doing it in the first place.^19^ Their solution was to use a temporal median filter—their interest was in single-molecule localization microscopy methods such as dSTORM and PALM, where the signals of interest (blinking fluorophores) are on for very few frames at a time. Thus, using a temporal median filter disregards background contributions that are on for many frames while keeping contributions from the single molecules. Ma et al. used a similar concept in their WindSTORM image processing program,^20^ specifically that of “extreme value based emitter recovery”, with their approach being more robust to denser emitter populations than the temporal median filter.^21^ Both methodologies assume fluorescence intermittency, or blinking, of fluorophores, and thus in experiments without blinking, they fail. Möckl et al. trained a deep neural network to subtract structured background from microscopy images;^18^ however, training such a neural net to anticipate large autofluorescent objects (from our experience imaging human brain tissue, such objects can occupy ∼500 × 500 pixels^2^) could be laboriously long. In their implementation, training on 12 × 12 pixel^2^ images took approximately 1 h; thus, scaling up to a 512 × 512 pixel^2^ image would suggest weeks of training. Another suite of approaches to get around the effect of autofluorescence includes hydrogel-based tissue transformation technologies, which are applicable to tissues but not to live cells. These, broadly speaking—for a detailed recent review, see Choi et al.^22^—aim to engineer tissue physiochemical properties while preserving the cellular and molecular spatial context. Tissue properties that can be engineered include optical transparency^23^ and tissue size.^24^ These methods are undoubtedly powerful; however, depending on the tissue, they can be complex to execute and time-intensive. For example, the OPTIClear protocol, optimized for human brain materials, can in total take from days to months from protocol beginning to imaging, dependent on the tissue.^25^

**Figure 1:**
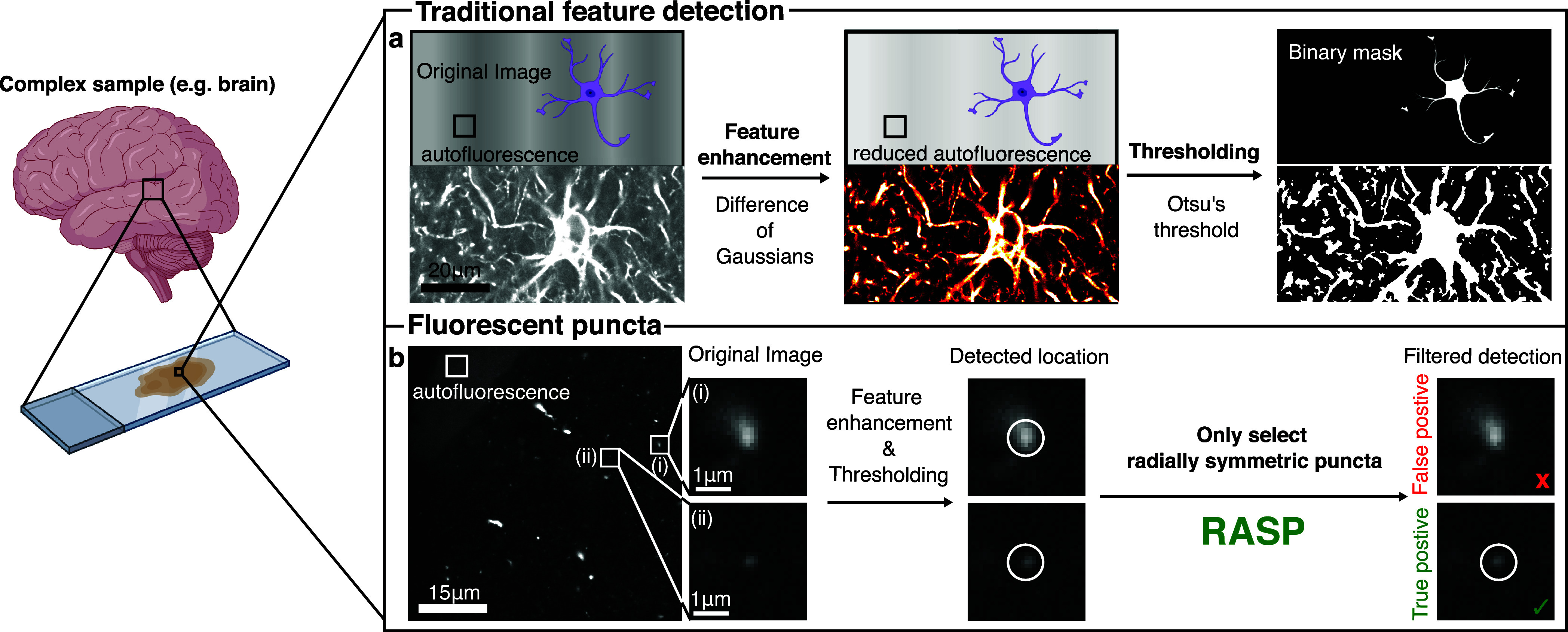
RASP enables accurate fluorescent puncta detection beyond the state-of-the-art. (a) Illustration of a conventional feature detection strategy composed of a feature enhancement step, e.g., a Difference-of-Gaussians filter,^10^ to accentuate the differences between the desired feature signal and background, and a thresholding step, such as Otsu’s method,^11^ that converts a feature-enhanced image to a binary mask. (b) In the presence of structured background, objects below the diffraction limit cannot be precisely detected by conventional feature detection strategies. RASP, an added selection step, distinguishes symmetric puncta, thus eliminating false positives. Elements of this figure were created with BioRender.com.

Furthermore, high-throughput imaging is increasingly needed to answer biological questions.^26^ This is due to the statistics needed to uncover small, biologically relevant effects—in the previously discussed example of Zhang et al., images of 300,000 cells were needed in their smFISH experiment to have the statistics necessary to firmly establish biological conclusions.^8^ In order to develop a similar picture of the whole mouse brain, the same group recently imaged approximately 7 *million* cells using FISH.^27^ Therefore, contemporary biology increasingly requires computationally efficient processes to match the increasing number of large data sets. In images of complex systems, traditional feature detection is able to accurately determine cell boundaries from single images containing structured background (Figure 1a). However, this is only half the battle. In detecting fluorescent puncta, structured background can appear extremely similar to a diffraction-limited spot (Figure 1b). How do we, with high precision and efficiency, distinguish between a false positive and a true positive in this context? Furthermore, once detected, can we use this single punctum information to determine the relative spatial statistics (i.e., density, extent of clustering, *etc.*) within segmented cell boundaries?

Inspired by the work of Parthasarathy,^28^ whose central insight was that the intensity of any imaged particle is radially symmetric about its center, as well as by the SRRF^29,30^ and SOFI^31^ techniques, we reasoned that using metrics based on the radial symmetry of a detected punctum may enable us to reject false-positive puncta detected due to structured background. Specifically, we reasoned that two metrics relating to radial symmetry could be used to reject false positives. One, which we term flatness, is calculated by comparing the local maximum intensity of a punctum to the intensity at its radius. We intuit that for true positives (which should be close in shape to 2D Gaussians and thus not flat), this value should be lower than that for false positives. The other metric, which we term integrated gradient, sums the gradient magnitude at a defined radius around a punctum, as we also intuit that for a symmetric punctum (a true positive), this metric will be larger than that for a nonsymmetric punctum. Based on Parthasarathy’s further demonstration that such an approach was computationally efficient, we also reasoned that our use of the radial symmetry would be fast and thus compatible with high-throughput imaging. We thus think that for structured background, our approach should be optimal. We term our approach RASP (Radiality Analysis of Single Puncta) and show, using simulations and experiment, that it enables the fast rejection of false positives in images containing structured background and that this should enable more precise correlations between cellular locations and fluorescent puncta in future work. We hope that this approach, integrated into experiments such as smFISH, protein colocalization experiments, and tissue imaging, can improve the repeatability and reliability of high-throughput imaging-based data sets.

## 2 Methods

### 2.1 Optical Setups

Experiments were performed on one of three microscopes: two widefield single-molecule microscopes (herein called “Microscope 1” and “Microscope 2”) or a spinning-disk confocal microscope (“Microscope 3”).

“Microscope 1” was a bespoke widefield fluorescence microscope, with the illumination entering the microscope body through the back illumination port, and has been described before.^32^ For completeness, the excitation path combined a 488 nm laser (iBeam-SMART, Toptica) and a 561 nm laser (LaserBoxx, DPSS, Oxxius). Each laser beam was circularly polarized using quarter-wave plates, collimated, and expanded to minimize field variation. These beams were aligned and focused on the back focal plane of the objective lens (100× Plan Apo TIRF, NA 1.49 oil immersion, Nikon) to enable highly inclined and laminated optical sheet (HILO) illumination. Fluorescence emission was collected using the same objective and separated from the excitation light by a dichroic mirror (Di01-R405/488/561/635, Semrock). Emission filters were used to further filter the emitted light (FF01-520/44-25 + BLP01-488R for 488 nm excitation; LP02-568RS-25 + FF01-587/35-25 for 561 nm excitation, Semrock). The filtered fluorescence light was expanded (1.5×) and projected onto an electron-multiplying charge-coupled device (EMCCD, Evolve 512 Delta, Photometrics) operating in frame transfer mode with an electron multiplication gain of 250 ADU/photon.

“Microscope 2” was a widefield fluorescence microscope (Eclipse Ti-E, Nikon), with the illumination entering the microscope body through the back illumination port, similar to a microscope described in Bruggeman et al.^33^ Specifically, in Bruggeman et al., it was described as Microscope 3. The beams from five lasers (Cobolt C-FLEX combiner with 405, 488, 515, 561, and two 638 nm lasers, free space) were coupled into a square-core multimode fiber (05806-1 Rev. A, CeramOptec) with a free space fiber launch system (KT120/M, Thorlabs). Speckles from the fiber were removed using a vibration motor, in a manner similar to the design of Lam et al.^34^ These beams were then focused to a spot in the back focal plane of an oil immersion objective (Plan Apo, 100 × 1.49 NA oil, Nikon) using an achromatic doublet lens (AC254-200-A, Thorlabs). This lens and a mirror were mounted on a linear translation stage (XR25C/M, Thorlabs) to allow manual adjustment of the beam emerging from the objective and switch between EPI, HILO, and TIRF illumination. The multimode fiber used for imaging negated the need for a quarter-wave plate as it achieved a highly randomized polarization at the sample plane. For imaging of the Tetraspeck beads, fluorescence was filtered by a dichroic beamsplitter (Di03-R405/488/532/635-t1, Semrock) and emission filters (BLP01-635R, Semrock). The fluorescence was focused on an sCMOS camera (Prime 95B, Teledyne Photometrics). A 4f system consisting of two achromatic lenses (AC254-075-A-ML and AC254-075-A-ML, Thorlabs) was included in the emission path, resulting in a total system magnification of 100× and thus a virtual pixel size of 110 × 110 nm^2^. The microscope PC was a Dell OptiPlex 7070 Mini Tower running on Windows 10 (64 bit), with an Intel i9-9900 processor and 32 GB RAM.

“Microscope 3” was a spinning disk confocal microscope (3i intelligent imaging). The microscope was equipped with a 200 mW, 488 nm laser (LuxX) and a 150 mW, 561 nm laser (OBIS). These lasers were housed in a beam combiner (3i intelligent imaging), which focused them into an optical fiber that sent the illumination light into a field flattener (Yokogawa-Uniformizer for CSUW). The excitation light was then passed into a spinning disk unit (50 μm-sized pinholes, Yokogawa CSU-W1 T2 Single Molecule Spinning Disk Confocal, SoRa Dual Microlens Disk) and then the microscope body (Zeiss Axio Observer 7 Basic Marianas Microscope with Definite Focus 3) using a dichroic mirror (FF01-440/521/607/700, Semrock). The fluorescence was filtered using either a FF01-525/45-25-STR filter (Semrock) in the case of 488 nm excitation or a FF02-617/73-25-STR filter (Semrock) in the case of 561 nm excitation. The fluorescence was then focused onto one of two sCMOS cameras (Prime 95B, Teledyne Photometrics). The objective lens was a Zeiss oil immersion objective (Alpha Plan-Apochromat 100*×*/1.46 NA Oil TIRF Objective, M27). The microscope was controlled using a PC (Dell-Acquisition Workstation 310R) and SlideBook software produced by the manufacturer (3i intelligent imaging).

### 2.2 Sample Preparation

#### 2.2.1 FFPE Human Brain Slices

Formalin-fixed paraffin-embedded (FFPE) tissue sections were obtained from the cingulate cortex (tables S2 and S3) and cut to 8 μm thickness. FFPE sections were baked at 37 °C for 24 h followed by 60 °C overnight. Sections were deparaffinized in xylene and rehydrated using graded alcohols. Nonspecific binding was blocked with 1% bovine serum albumin (BSA) solution in PBS for 30 min. The tissue was then pressure cooked in citrate buffer at pH 6 for 10 min. Tissue sections were incubated with primary antibodies: antiphosphorylated α-synuclein (ab184674, Abcam, 1:500; ab59264, Abcam, 1:200); Microtubule-Associated Protein 2 (ab254143, Abcam, 1:500); and ionized calcium-binding adapter molecule 1 (Wako-019-19741, FujiFilm, 1:1000) for 1 h at room temperature. The sections were then washed three times for 5 min in PBS followed by the corresponding AlexaFluor secondary antibodies [antimouse 568—A11031 (Thermo Fisher), antirabbit 568—A11011 (Thermo Fisher), antimouse 488—A11001 (Thermo Fisher), and antirabbit 488—A11008 (Thermo Fisher), all at 1:200] for an additional hour at room temperature in the dark. Sections were then washed three times for 5 min again in PBS and incubated in Sudan Black (0.1% for 10 min, 199664-25G, Sigma-Aldrich). Removal of Sudan Black occurred with three washes in 30% ethanol (E7148-500 ML, Sigma-Aldrich) before mounting with VECTASHIELD PLUS (Vector Laboratories, H-1900) and coverslipping (VWR, 50 × 24 mm #1 thickness, catalogue no. 48404-453) for imaging. Sections were stored at 4 °C until imaging was completed.

#### 2.2.2 TetraSpeck Experiments

Glass coverslips (Fisher Scientific, 12373128, #1 thickness 22 mm × 50 mm) were plasma cleaned for 30 min (Ar plasma cleaner, PDC-002, Harrick Plasma). An imaging chamber was created on the coverslips using Frame-seal slide chambers (9 × 9 mm^2^, SLF0201, Biorad) and coated with 0.01% w/v poly-l-lysine (PLL, P4707, Sigma-Aldrich). After excess PLL was removed and the samples were washed with filtered (0.02 μm syringe filter, Whatman, 6809-1102) PBS, a 1:625 stock dilution of 0.1 μm diameter fluorescent microspheres (TetraSpeck Microspheres, 0.1 μm, fluorescent blue/green/orange/dark red, Thermo Fisher, catalogue no. T7279) was added. These were then imaged on Microscope 2, i.e., a widefield single-molecule fluorescence microscope, with 488 nm excitation. The power density at the sample plane was 100 μW·cm^–2^ for the 488 nm excitation.

### 2.3 Simulation

**Figure 2:**
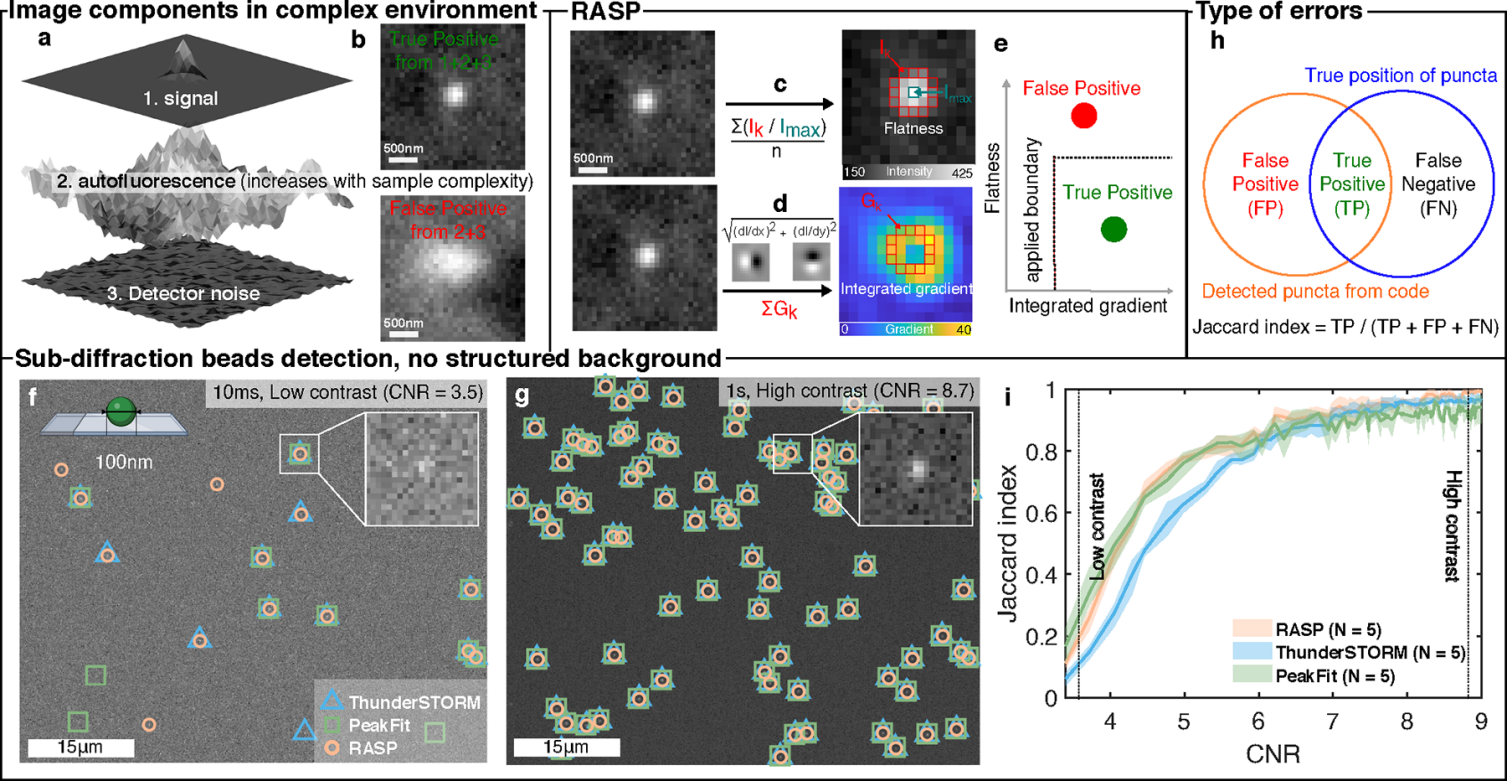
RASP distinguishes puncta by flatness and integrated gradient. (a) Images of complex samples are composed of signal, detector noise, and autofluorescence, which reduces the detectability of the signal of interest. (b) The measured pixel intensities for true positives (TPs) are the summation of detector noise, autofluorescence, and signal, whereas false positives (FPs) arise from autofluorescence and detector noise only. (c) Pictorial representation of the flatness calculation procedure using eq 6. (d) Pictorial representation of the integrated gradient calculation procedure using eqs 9 and 10. (e) FPs and TPs plotted by their flatness and integrated gradient, separable by a decision boundary. (f,g) Images of 100 nm diameter fluorescent beads were recorded with differing exposure times to capture low (10 ms) and high (1 s) contrast-to-noise ratios. Peaks were identified using RASP, ThunderSTORM, and PeakFit. (h) Illustration of the possible error types: false positives (FPs) are points wrongly detected, and false negatives (FNs) are undetected correct points. (i) Jaccard index comparison of RASP, ThunderSTORM, and PeakFit for five different fields of view (FOVs) where the ground truth was determined from the highest CNR image. Elements of this figure were created with BioRender.com.

Simulations were used to add simulated diffraction-limited aggregates (puncta) and large aggregates to the real negative control data. This real negative control data formed the structured background and was made up of 136 images of FFPE human brain slices where no primary antibody was added, but secondary antibody was still present. These images thus should contain only autofluorescence and detector noise (Figure 2). For large aggregates images from Parkinson’s disease patients, FFPE brain slices stained for α-synuclein were analyzed by hand. Regions-of-interest (ROIs) containing large aggregates from these images were cropped, and these cropped ROIs were saved in a “large aggregate library”. 100 manual selections were made from these images and added to the large aggregate library. For the diffraction-limited aggregates, a blank image with the same size as a negative control image was initially generated. 2D Gaussian-distributed puncta *g*(*x*,*y*) were simulated using
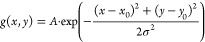
1

In this equation and those that follow, *x* and *y* are pixel indices in the *x* and *y* directions, and *x*_0_ and *y*_0_ are the origin coordinates in *x* and *y* of the 2D Gaussian. *A* is the amplitude per punctum (set as the same for every punctum in our simulations), and σ is the punctum width. The punctum widths were microscope-type dependent, with us using a σ of 1.4 for confocal imaging and σ = 1.2 for widefield imaging. These puncta were then added in a grid-like arrangement onto the blank image. The σ value was determined by taking images using the protocol of Section 2.2.2 but on Microscope 1 (i.e., a widefield single-molecule fluorescence microscope) and Microscope 3 (i.e., a spinning-disk confocal microscope) described in Section 2.1. The 561 nm laser was used for excitation, the same excitation wavelength used for imaging aggregates in human brain tissue. A binary mask was generated alongside a simulated punctum image to denote the position and area covered by each punctum. This binary mask was generated using Otsu’s thresholding method^11^ applied to the simulated spot image. This process was repeated by changing the intensity per punctum, and simulated punctum images at different intensities were saved in the diffraction-limited punctum library.

To add large aggregates onto the background (i.e., negative control images), a randomly cropped ROI was chosen from the large aggregate library. Otsu’s threshold was then applied to the ROI determining the position of aggregate (1 in the resultant binary mask) and background (0 in the resultant binary mask). The binary mask was converted to a distance matrix by the bwdist function in MATLAB for each background pixel. The function calculates the Euclidean distance between a background pixel and its nearest aggregate pixel.

Subsequently, a sigmoid function *c*(*x*,*y*) was calculated using the following equation
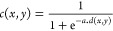
2where *d*(*x*,*y*) is the value in the distance matrix, *a*, the scaling factor, was 10 for the simulation, and *c*(*x*,*y*) was the resulting correction value for each pixel. The ROI, *I*_ROI_(*x*,*y*), was then multiplied by the correction value *c*(*x*,*y*) from eq 2 to minimize the structured background in the cropped image while keeping only the signal from the large aggregate.
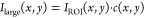
3*I*_large_(*x*,*y*) was zero-padded to be the same size as the negative control image. The zero-padding length was random in each direction, adding the large aggregate to a random position within the image. For diffraction-limited aggregates, a simulated punctum image *I*_sim_punctum_(*x*,*y*) with a specified intensity was first run through a Poisson random number generator to generate a more realistic simulation, *I*_punctum_(*x*,*y*).
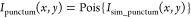
4

Finally, the simulated image *I*_sim_(*x*,*y*) was generated by adding the background image *I*_bg_(*x*,*y*), the simulated punctum image with Poisson noise *I*_punctum_(*x*,*y*), and large aggregate image *I*_large_(*x*,*y*) together using
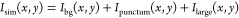
5

The background per diffraction-limited aggregate was determined by the mean value of the background covered by this aggregate (i.e., the area of 1*s* on the binary mask per aggregate). The sum intensity was determined by the sum value of the simulated punctum image covered by this aggregate, and the CNR was determined by the difference between the signal maximum and the mean of the background, which was then divided by the standard deviation of the background, as described in eq 11. Finally, any diffraction-limited aggregates overlaid with large aggregates were deleted.

### 2.4 Camera Gain Calibration

To convert the pixel value to photons in an sCMOS camera, we recorded a series of image sequences at seven different intensity levels (1000 frames per intensity level) with uniform illumination, including one level at no illumination for the calculation of camera offset. For every pixel, the mean and variance were calculated across the 1000 frames, generating seven different variance and mean values corresponding to the seven nonzero illumination intensities. The camera offset per pixel was determined as the mean pixel value in the nonilluminated frame. The camera gain per pixel, expressed in photoelectrons per count, was determined by calculating the slope between the seven variance and mean values per pixel and subtracting the nonilluminated frame offset.^35^ Software available for this purpose is available at https://doi.org/10.5281/zenodo.10475643.

### 2.5 Puncta Detection Method with RASP Filtering

Images underwent a high-pass kernel, obtained through the difference between the original image and a Gaussian-blurred image (σ = 1.4_px_), followed by a Laplacian-of-Gaussian^36^ (LoG) kernel (σ = 2_px_), which is the second spatial derivative of a 2D Gaussian distribution, for punctum feature enhancement. Thresholding involved selecting the top 5% brightest pixels from the processed image and converting them to 1, while the remaining 95% were assigned a value of 0. For each object in the binary mask, the flatness and integrated gradients were calculated from the original image. Next, all binary objects were filtered by their flatness and integrated gradient with a boundary determined from a negative control image. The code was run on a Dell Precision 3650 PC with an Intel i9-11900 processor and 80 GB of RAM.

### 2.6 Analysis of Data Using PeakFit

The PeakFit macro of GDSC SMLM^37^ (version 1) was used for batch processing data utilizing a “Circular Gaussian 2D” for punctum detection in both bead and brain images. Camera gain was set to be 1, and offset was set to be 0. In the bead experiment, a “single mean filter” with “relative smoothing” set at 1.4 and default parameters for “search width”, “border width”, and “fitting width” were utilized. Default settings for “shift factor” and “signal strength” were applied, with the “minimum photons” set to 10 and the “minimum and maximum width factors” set to 0.54 and 2, respectively. For brain images, a “difference Gaussian filter” was employed with “smoothing” parameters set at 0.7 and 2.5 for “smoothing2”. The Spot Finder, a core component of PeakFit, was employed to manually select the acceptance ratio of detected puncta. Puncta with the top 3.5% intensity were used in the wide-field imaging simulation, while those with the top 5% intensity were used in the confocal imaging simulation. The code was run on a Dell precision 3650 PC with an Intel i9-11900 processor and 80 GB RAM.

### 2.7 Analysis of Data Using ThunderSTORM

Version 1.3 of the ThunderSTORM macro^38^ was used for batch processing of the data. Puncta detection in both bead and brain images involved a “wavelet filter (B-Spline)” with scale 2.0 and order 3, followed by “nonmaximum suppression”. For bead data, a threshold of 1.1 times the standard deviation of “wave.F1” was applied. In simulated brain images in both widefield and confocal imaging modes, a threshold of 0.6 times the standard deviation of “wave.F1” was utilized. No estimator or renderer was employed in this process. The code was run on a Dell precision 3650 PC with an Intel i9-11900 processor and 80 GB RAM.

## 3 Results and Discussion

Fluorescence images of tissue and cells can be described as being composed of three distinct components: signal, autofluorescence, and detector noise (Figure 2a). A true-positive punctum, i.e., the signal we wish to detect, is composed of all three components (Figure 2b)—a false positive is composed only of autofluorescence and detector noise. The difficulty arises in that true and false positives can look extremely similar. To address this challenge, we propose RASP, which, in essence, is a filtering step after puncta detection where false positives and true positives are distinguished based on their radial symmetry or “radiality”. We quantified the radiality of individual puncta using two metrics: flatness (Figure 2c) and integrated gradient (Figure 2d). Flatness is defined as the mean ratio between intensity values at all pixels contained within a ring of pixels 2 pixels away from the local maximum (*I*_*k*_), where *k* represents the *k*th pixel in the set of pixels at radius 2 pixels distance (Figure 2c) and intensity values at the local maximum (*I*_max_). This radius is related to the optimal trade-off between point-spread function sampling and maximizing signal above background in single-molecule spectroscopy. Using theory and simulations, Thompson et al.^39^ showed that in order to sample individual fluorescent probes optimally, the PSF should be around twice the pixel size—thus elucidating our choice of a 2 pixel radius, and that it should be generally applicable to single-molecule microscopes. The value corresponds to the outer radius of a single fluorescent punctum and can also be derived from experiment: it is the nearest integer pixel value to the half-width-half-maximum of our point spread function (see Figure S8. Such a value would change depending on optical implementation (and strictly speaking, wavelengths imaged), but for optimal single-molecule imaging, it should always be around 2 pixels. It can however be calibrated in the RASP software for optimal use on a specific microscope. The flatness is calculated using eq 6
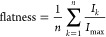
6where *n* is the number of pixels and *k* refers to the *k*th pixel. The integrated gradient is the sum of gradient values (*G*_pixel_^*k*^) from all pixels contained within a ring of pixels 2 pixels away from the local maximum, where *k* represents the *k*th pixel (Figure 2d). To compute this, the gradient fields in the *x* and *y* directions, *G*_*x*_(*x*,*y*) and *G*_*y*_(*x*,*y*), are first calculated from the original image *I*(*x*,*y*) using
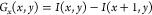
7where *x* and *y* refer to pixel indices in the *x* and *y* directions, and
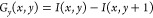
8*G*, the gradient magnitude, is then calculated using eq 9
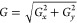
9and then, the integrated gradient is calculated using eq 10
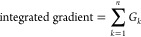
10

Subsequently, the flatness and integrated gradient values of the detected puncta are used to filter out false positives using a decision boundary (Figure 2e), which we determine using negative control experiments, as discussed further in what follows.

We first evaluated RASP in an ideal scenario, without any structured background, i.e., a situation where both RASP and existing state-of-the-art codes should perform well. To do this, we imaged bright, 100 nm diameter fluorescent beads (0.1 μm Tetraspeck Microspheres, Thermo Fisher) excited with 488 nm light using a widefield single-molecule fluorescence microscope (“Microscope 2”, section). Five different fields of view (FoVs) were imaged, with each FoV containing 100 frames of 10 ms per frame. This on average leads to ∼75 photons per punctum in one frame, meaning we can generate images of very low photon flux to relatively high (∼7500 photons per punctum after averaging 100 frames) photon flux. The conversion from counts to photons can be found in the Methods section. We then used these data to evaluate the code’s performance under different contrast-to-noise ratios (CNRs). The CNR is an image quality metric, defined as the contrast between the signal maximum (*S*_A_) and background (*S*_B_) divided by the standard deviation of the background (σ_B_)
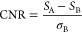
11

The CNR was controlled by integrating different numbers of frames from a static fluorescent bead sample, thereby achieving different CNR levels from an identical FoV. We conducted performance comparisons between RASP (Methods section for an overview of the punctum detection algorithm), PeakFit^37^ (Methods section), and ThunderSTORM^38^ (Methods section). PeakFit was chosen as it has been shown, for images that are not too densely filled with fluorescent puncta, to perform the best in a recent test of single-molecule punctum-detection codes.^40^ ThunderSTORM was selected as one of the most widely used punctum identification codes. Two example images illustrating low (Figure 2f) and high (Figure 2g) CNR regimes are shown, where the functional output from all three codes at high CNR shows 100% coincidence. Thus, these detection locations served as our ground truth positions for the characterization of code performance at lower CNR. The Jaccard index (Figure 2h), the true detected locations divided by the size of the union of detected locations and ground truth locations, was measured at a range of CNR values. Sensitivity and precision were also measured, and these are shown in Supplementary Note S1. Notably, RASP performed as well as PeakFit here, i.e., as well as the state-of-the-art. This experiment thus shows that RASP performs well at detecting puncta in images without a structured background.

**Figure 3:**
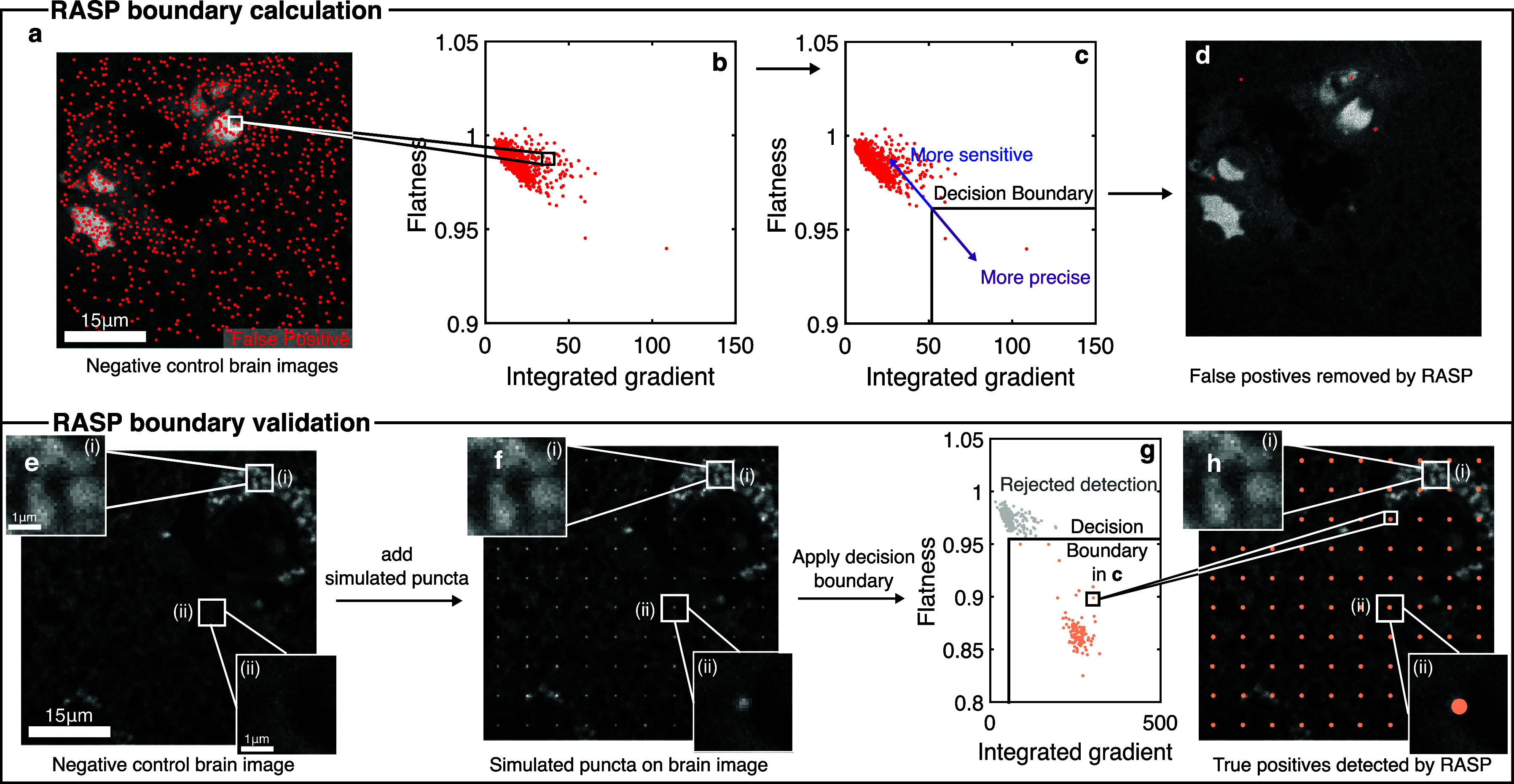
Implementation of RASP. (a) Detected puncta in a negative control FFPE brain tissue sample lacking primary antibodies but still containing secondary antibodies. (b) Flatness and integrated gradient values for the peaks in (a). (c) Determination of a decision boundary based on the flatness and integrated gradient for all detected puncta. (d) Filtered puncta within the decision boundary. (e) Negative control brain images with two zoomed-in regions. (f) Real brain images with added simulated diffraction-limited puncta (see the Methods section). (g) Scatter plot of all detected puncta in (f) with the decision boundary determined in (c). (h) Filtered detected puncta for the real brain image with added simulated puncta.

We now discuss how to use RASP to reject the false positives that arise when imaging complex systems. RASP implements this filter as a decision boundary (Figure 2e), which is generated using the negative control images that are taken routinely as part of any experiment. We have tested RASP using an exemplar of a complex system containing structured background, specifically FFPE human brain slices from patients with advanced Parkinson’s disease, stained with primary and secondary antibodies for α-synuclein and multiple cell types (see Methods, Section 2.2.1). These samples represent exemplars of samples containing complex, structured background and also of the sample types that quantitative microscopy increasingly studies—samples where the spatial organization of proteins, and/or single RNA/DNA molecules, relative to cells is of great interest. Thus, doing accurate cellular segmentation and accurate puncta detection of these samples is vital. Negative control images here were brain slices containing no primary antibody (but still stained with secondary antibody), imaged using the same spinning-disk confocal microscope (Microscope 3, Section 2.1). In order to determine the decision boundary, the flatness and integrated gradient values of puncta detected in the negative control images were used—these detected puncta can be assumed to be false positives (Figure 3a). The flatness and integrated gradient values for these puncta (Figure 3b) are then used to calculate the decision boundary. Boundaries are determined for flatness and integrated gradient separately (Figure 3c) and are typically set to be at the top 5% in the two dimensions separately. This parameter is a user-controlled parameter, however, and can be made more stringent at the penalty of losing some true positives. Applying this decision boundary to the same negative control data resulted in Figure 3d.

To illustrate the implementation of such a trained boundary, and its ability to distinguish between true and false positives, we added simulated diffraction-limited puncta to real negative control brain images and used RASP to analyze these new “real + simulated” images. The simulated diffraction-limited puncta (σ = 1.4, CNR = 8.7) were first run through a Poisson random number generator, to simulate shot noise, and then added onto the negative control image (Figure 3f, see Methods section). RASP’s feature enhancement and punctum detection process, detailed in Section 2.5, was then applied to these images. Analogous to the previous steps (Figure 2b), the flatness and integrated gradient values for all detected locations were calculated. Subsequently, the boundary established earlier by using negative control images (Figure 3c) was applied (Figure 3g). The resultant filtered puncta locations showed excellent coincidence with the simulated locations (Figure 3h), showing the power of RASP in removing false positives and keeping true positives. More detailed validation of this boundary selection method is shown in Supplementary Note S2. As an aside, we also provide an accurate method, alongside RASP, to estimate the intensity and background per detected puncta in structured background data, with a greater computational efficiency compared to that of the typical Gaussian fitting method—in our case, we find a ∼360× speed-up relative to Gaussian fitting; see Supplementary Note S9 for further details.

To compare the performance of RASP to the state-of-the-art methods in detecting puncta in images with structured background, i.e., images of cells or tissue, we imaged primary and secondary antibody-stained FFPE human brain slices from Parkinson’s disease patients at advanced stages of the disease. Specifically, we stained for α-synuclein, a protein responsible for the pathological hallmarks of Parkinson’s disease (PD)—aggregates of this protein are found in human brain regions at different sizes depending on disease severity.^41^ In particular, oligomeric aggregates that are smaller than the diffraction limit of light have been heavily implicated in disease pathology,^42,43^ with Emin et al. recently finding small, sub-100 nm oligomeric species found in PD brains to be far more toxic than the larger aggregates typically found in control brains.^44^ More recently, Matsui et al. demonstrated that a novel phosphorylation of the α-synuclein protein led to oligomer formation and that this led to cell death and neurodegeneration in their zebrafish models.^45^ This thus motivates the finding of puncta in images stained for α-synuclein as these puncta report on the presence of small oligomeric species that are otherwise difficult to detect and pathologically significant.

**Figure 4:**
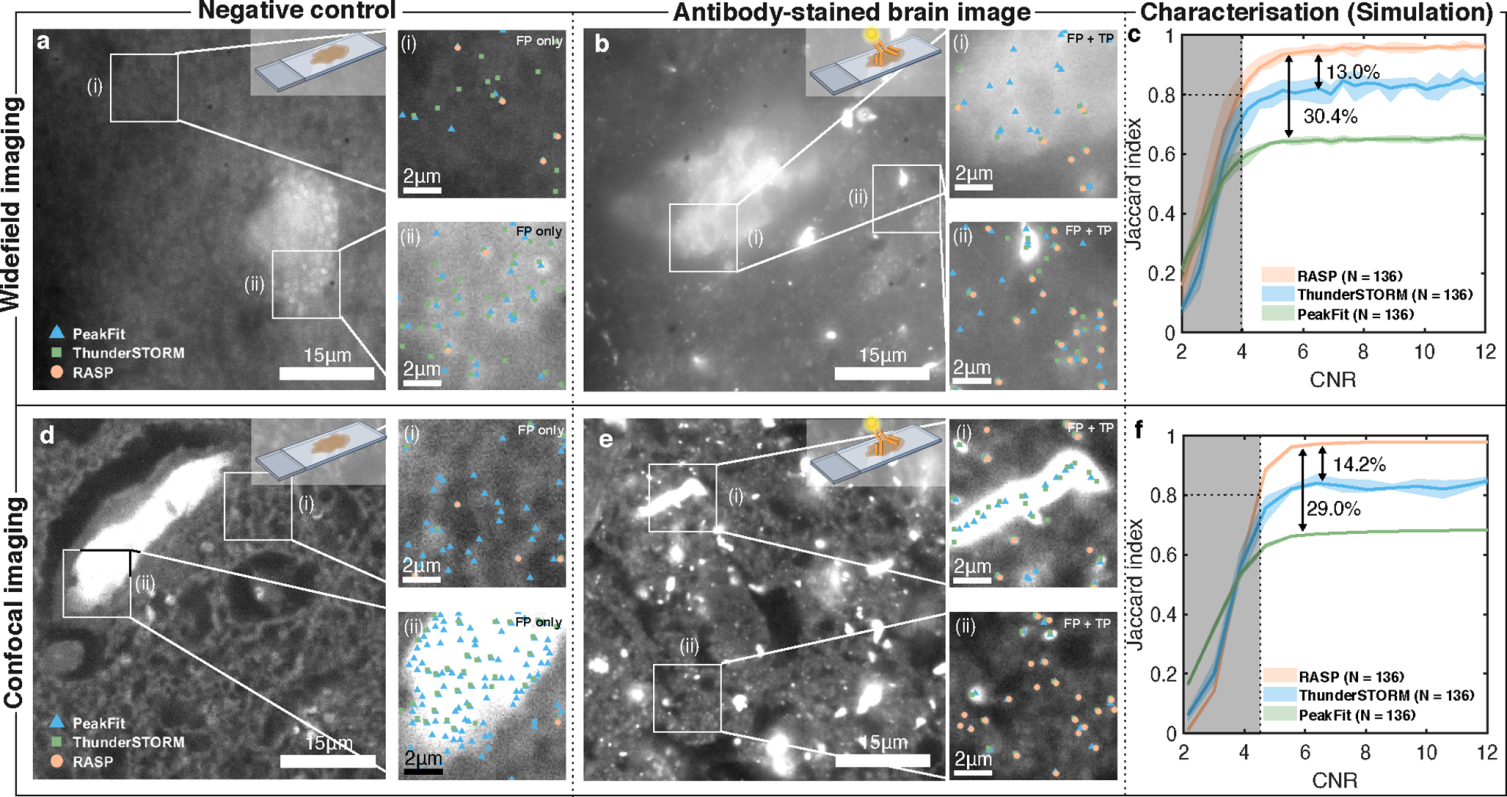
RASP outperforms traditional punctum detection in images with structured background. (a,d) Negative control FFPE brain slices imaged in widefield and confocal imaging modes, respectively, with two zoomed-in sections illustrating false positives from PeakFit, ThunderSTORM, and RASP. NB that these two different imaging modes correspond to two different microscopes. (b,e) α-Synuclein-antibody stained FFPE brain slice imaged in widefield and confocal imaging modes, respectively, with zoomed-in sections comparing the performance of PeakFit, ThunderSTORM, and RASP. (c,f) Jaccard index characterization for widefield imaging and confocal imaging modes, respectively, of PeakFit, ThunderSTORM, and RASP on real images of negative control FFPE brain slices with simulated puncta added. 136 real brain images with simulated puncta added were used for the characterization of each of the widefield and confocal imaging modes. Elements of this figure were created with BioRender.com.

We imaged these FFPE human brain slices with Microscope 1 (a widefield single-molecule fluorescence microscope) or Microscope 3 (a spinning-disk confocal microscope) (Section 2.1) to detect oligomeric aggregates of α-synuclein. We randomly selected 20 negative control images from a pool of 136 and used these in the same procedure as shown before (Figure 3c) to determine the decision boundary for the RASP filtering. We then applied this boundary to the remaining negative control images (Figure 4a,d) to further demonstrate how well RASP performed. As is clearly visible in Figure 4a,d, RASP far outperforms PeakFit and ThunderSTORM in the rejection of false positives from the negative control images. In fact, PeakFit and ThunderSTORM heavily overlabel the negative control images and structured background, which RASP’s filtering step avoids. This same boundary then was applied to images of FFPE brain slices stained for α-synuclein (Figure 4b,e). Notably, ThunderSTORM and PeakFit exhibited greater susceptibility to structured background and large features within the images, meaning that these codes will always overlabel an image of a complex system and thus detect a large number of false positives. By contrast, more than 90% of the puncta detected by RASP were colocalized with puncta detected by ThunderSTORM and PeakFit, while rejecting the false positives from larger objects and structured background. This shows that RASP simultaneously preserves the detection sensitivity and significantly increases the precision of true puncta detection. A gallery of true-positive and false-positive images, highlighting that it is the combination of flatness and integrated gradient that is necessary to distinguish the true and the false positives, from α-synuclein-antibody-stained FFPE human brain slices is shown in Supplementary Note S4.

To validate the performance of RASP on images with structured background and large features, we used images from both Microscopes 1 and 3 (Section 2.1) of FFPE brain slices containing no primary antibody but still stained with secondary antibody, with added simulated diffraction-limited puncta (see Section 2.3). Validation using images of primary and secondary stained FFPE brain slices was deemed to be both too subjective and too labor-intensive for manual annotation, given the substantial number of puncta across multiple images. To mitigate these challenges, we utilized 136 biologically negative control images from both widefield and confocal imaging. For each negative control image, we added 4 or 30, dependent on whether the image was widefield or confocal, randomly oriented large aggregates, drawn from a library of manually selected large aggregates from widefield and confocal images. Additionally, 400 or 1600, dependent on whether the image was widefield or confocal, randomly distributed diffraction-limited puncta were overlaid on the widefield and confocal images, the number of which was determined to match the real aggregate density. Then, a series of simulated images were generated with the puncta at the same positions but with different intensities, yielding a range of CNRs from 2 to 12.

For high CNR widefield images, the Jaccard index reached 95.0 ± 0.5%, 82.1 ± 1.9%, and 65.0 ± 0.4% for RASP, ThunderSTORM, and PeakFit, respectively (Figure 4c). For high CNR confocal images, the Jaccard indexes were 97.8 ± 0.2%, 83.7 ± 1.9%, and 68.7 ± 0.18% for RASP, ThunderSTORM, and PeakFit, respectively (Figure 4f). Graphs showing precision and sensitivity can be found in Supplementary Note S3. This shows that RASP outperforms two state-of-the-art codes when it comes to precisely and sensitively detecting puncta in structured background environments: essential for high-throughput imaging needed in modern biological experiments. Further, within 136 negative control images, the number of false positives detected was 53 ± 42, 1278 ± 497, and 1716 ± 41 for RASP, ThunderSTORM, and PeakFit, respectively (Supplementary Note S3). This demonstrates RASP’s capacity to effectively distinguish true puncta from false positives while maintaining a similar sensitivity performance, as at high CNRs, the sensitivity of all three codes is identical. Therefore, RASP can precisely detect fluorescent puncta in the presence of structured backgrounds in images of real, complex, biological systems. Furthermore, as RASP is a filtering method, by calculating the flatness and integrated gradient, and using the same decision boundary, for the ThunderSTORM- and PeakFit-detected puncta, there is a significant increase in precision with minimal decrease in sensitivity for both ThunderSTORM and PeakFit (Supplementary Note S5). This serves to further highlight that the RASP filtering step, being computationally efficient and data-driven, is a general step that can be added after more sophisticated punctum identification codes and other codes in the future. This shows a detection method that should heavily speed up the analysis of high-throughput protein, DNA, and RNA colocalization experiments that seek to answer biological questions that require large statistics.

**Figure 5:**
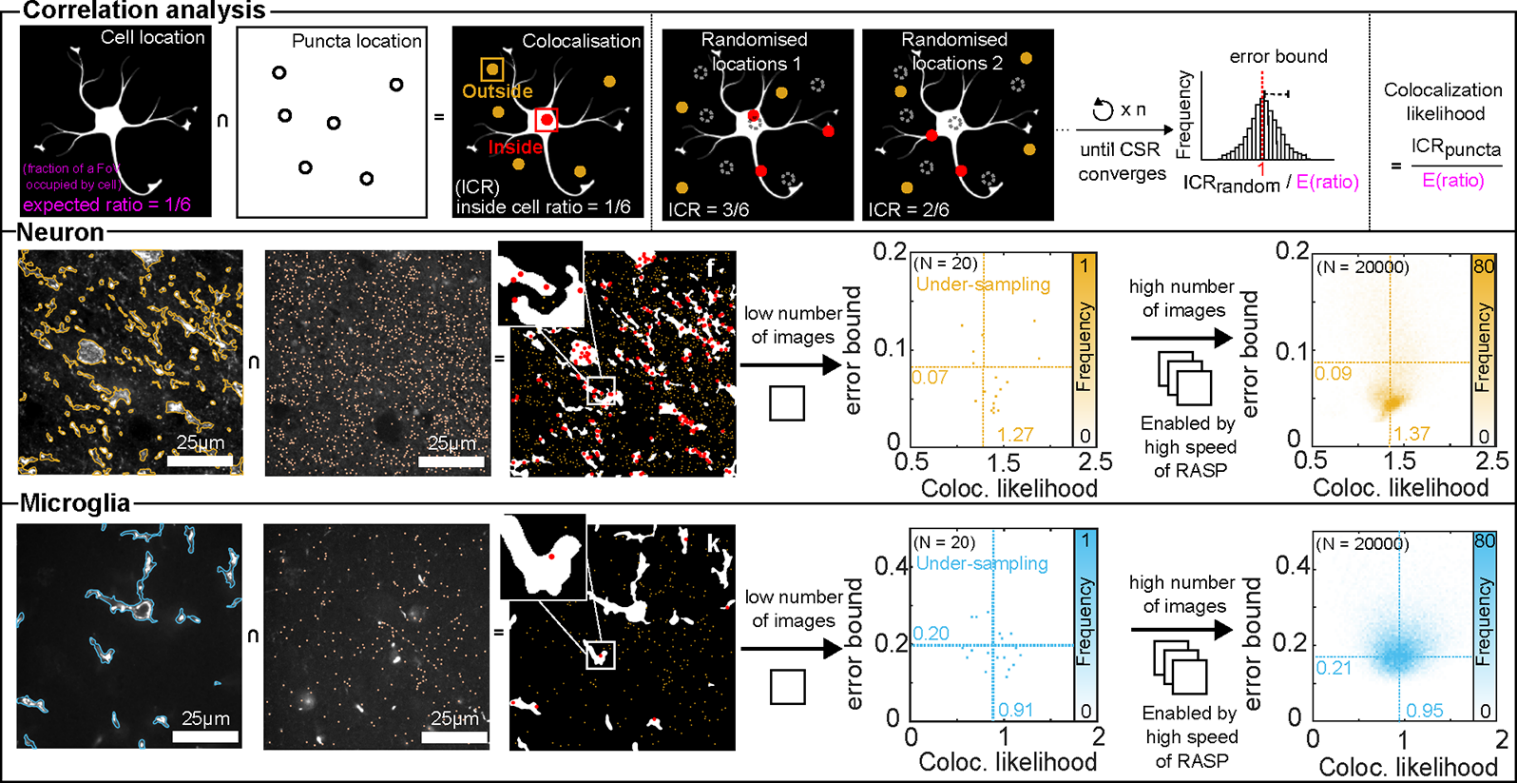
Correlation analysis between cell and fluorescent puncta. (a) ICR calculation between cells and detected puncta, i.e., the number of inside locations divided by the total number of locations. (b) ICR between cells and a random spatial distribution of puncta, referred to here as CSR data. The number of puncta is chosen as identical to the number in real data. (c) Formula for calculating the colocalization likelihood between cells and puncta, where we compare the ratio of puncta found inside cells to what would be expected if puncta were distributed uniformly—the fraction of the image occupied by cells. The colocalization likelihoods of CSR data should converge to 1 if enough locations have been sampled. These data are then used to calculate the error bound on the colocalization likelihoods. (d,i) Overlapping detected neurons and microglia locations, respectively, with the original image. (e,j) Detected puncta locations in the original image. (f,k) Inside puncta (red) and outside puncta (yellow) based on cell locations. (g,l) Colocalization likelihood distribution with 20 FOVs used. (h,m) Colocalization likelihood distribution with 20,000 FOVs used. Elements of this figure were created with BioRender.com.

Finally, we demonstrate that RASP’s high precision, sensitivity, and computational speed enables a high-throughput analysis of the correlation between various neuronal cell types and α-synuclein aggregates in the human brain directly, which could aid our understanding of the important role of α-synuclein in cellular toxicity—a role that remains incompletely understood.^46^ To measure these correlations, we initially eliminated all out-of-focus images using an automated procedure described in Supplementary Note S6 and then analyzed the remaining images. For the diffraction-limited aggregates, the inside cell ratio (ICR) was computed as the ratio between the number of puncta inside the cell over the total number of puncta per FoV (Figure 5a). This was then compared to what we would expect if the puncta were distributed normally over the image—i.e., the fraction of the image occupied by the cell mask. This enables us to calculate a quantity we refer to as the colocalization likelihood—the ratio between the ICR of real puncta locations and the ICR of random puncta locations (Figure 5c). This likelihood provides a measure of whether we are more likely to find an α-synuclein aggregate inside or outside of a particular cell type in comparison to a random distribution. We also compute this likelihood ratio with randomized positions of an identical number of puncta, data we refer to as “complete spatial randomness” data (CSR). These data, when a sufficiently large number of random positions have been sampled, should converge to 1 when divided by the fraction of the image occupied by the cell mask. When this quantity has converged (see Supplementary Note S8 and Figure S9), we use the CSR data to give an error bound on the colocalization likelihood ratio of a single image, Figure 5b.

RASP’s high-throughput nature enabled us to conduct a likelihood analysis for neurons and microglia, utilizing 20,000 FOVs from three PD cases in the ACG for each cell type, covering dimensions of 3.96 mm × 3.96 mm × 12 μm per patient—approximately 750 GB of image data. In the case of randomly selecting 20 FOVs, the colocalization likelihood derived from aggregate data was 0.91 ± 0.20 for microglia and 1.27 ± 0.12 for neurons, while the error bound, computed using CSR data, on these colocalization likelihoods was 0.20 ± 0.05 for microglia and 0.07 ± 0.05 for neurons (Figure 5g,l). However, it is clear from examining the histograms in Figure 5g,l that we have not sufficiently sampled our colocalization likelihood space—the histograms are sparse, and it is unclear if the mean and standard deviations are genuine or a result of low amounts of data. As RASP enables high-throughput data analysis, analyzing the entirety of the 20,000 FOVs shows that as we include more data, the mean value from the aggregate data converged. Specifically, the colocalization likelihood from the aggregate data was 0.95 ± 0.28 for microglia and 1.37 ± 0.32 for neurons, while the error bound, computed using CSR data, of these likelihoods was 0.21 ± 0.06 for microglia and 0.09 ± 0.06 for neurons (Figure 5h,m). It is also clear in Figure 5h,m that these standard deviations and means are truly representative of the data—we are no longer sparsely sampling our colocalization likelihood, and thus, we have enabled robust biological conclusions. The results that neurons are more likely to contain α-synuclein aggregates align with findings from other papers that aggregates are more likely to be inside neurons and aligns with the hypothesis that it is in neurons that these aggregates grow.^46−48^ Importantly, we, for the first time and enabled by RASP, can observe these correlations between aggregates smaller than the diffraction limit and neurons in human brain slices.

## 4 Conclusions

We have in this work introduced RASP, a method that uses flatness and gradient information of isolated fluorescent puncta to increase the precision of puncta detection in microscopy experiments without a loss of sensitivity. The method relies on the symmetrical shape of a fluorescent punctum in order to reject other detected puncta that are not. Our hope is that by improving this false-positive rejection, RASP can form a valuable step that increases analysis reliability in high-throughput biological experiments involving the imaging of complex cellular systems. We also demonstrate that RASP does not require laborious simulations or additional experiments to work effectively: the discriminator that rejects false positives is learned from negative control data that would be taken as part of a typical experiment.

We have demonstrated that RASP performs well on both images without structured background (Figure 2) and that RASP’s true-/false-positive rejection boundary, learned from negative control data, reliably distinguishes between true and false positives in situations with structured background (Figure 3). We show that it outperforms state-of-the-art puncta detection codes in images with structured background (Figure 4) and thus, for the analysis of these images, provides a valuable tool to enhance the precision of puncta detection with no loss in sensitivity. As RASP’s filtering step comes after an initial detection of puncta in an image, we have also shown that it improves the precision of puncta detection, with no sensitivity loss, when combined with other puncta detection codes (Supplementary Note S5). This, coupled with its computational efficiency—it requires approximately 30% of the time required by ThunderSTORM to process a 1200 × 1200 pixel^2^ image in our tests—demonstrates that RASP can be a simple filtering step added to the analysis of high-throughput imaging data to improve analysis precision. We also note that these experiments have been conducted across multiple instrument types, widefield and spinning-disk confocal microscopes (Figure 4), and thus, RASP should be generally applicable in fluorescence imaging—only negative control images are needed.

Understanding biological systems increasingly demands the extraction of the most information from the fewest images of the largest area at the highest feasible resolution. We show, in Figure 5, that RASP enables this—we were able to use this code to determine the likelihood of finding a protein aggregate colocalized with a cell across 20,000 FOVs, enabling biological conclusions from large data sets. This highlights RASP’s relevance for protein/RNA/cell colocalization experiments, such as FISH, where large numbers of cells are increasingly needed to be imaged to understand biological effects. Imaging of 300,000 cells^8^ or 7 million cells^27^ in tissues demands strategies that can quickly, using single images, distinguish between structured background and real fluorescent puncta we wish to analyze. We show that RASP adds a tool to do this that does not require laborious sample preparation or time-intensive simulations for background reduction. We anticipate its use in high-throughput single-molecule experiments and also that in the future, the implementation of more advanced decision boundaries will improve RASP’s performance.

## Supporting Information Available

Precision and sensitivity for bead detection, validation of the boundary selection method, precision and sensitivity on puncta detection on human brain tissue, gallery of puncta detected by RASP, RASP combined with ThunderSTORM and PeakFit, RASP’s procedure for rejecting out-of-focus images, procedure for determining the diffraction-limited area threshold, procedure for colocalization likelihood ratio bootstrapping, intensity and background estimation method validation, data on RRID, patient information , and staining plan. In addition, detailed protocols can be found in support of this study on protocols.io. Specifically: Tetraspeck Experiments: https://doi.org/10.17504/protocols.io.4r3l22br4l1y/v2 FFPE Human Brain Slices: https://doi.org/10.17504/protocols.io.5qpvorp6bv4o/v2 Codes and data in support of this study can be found in the following locations: RASP code (v1), raw data, ThunderSTORM v1.3 and GDSC SMLM v1 installers and codes, along with raw data for all figures except Figure 5 may be found at: https://doi.org/10.5281/zenodo.10407297. The GitHub for the MATLAB code may be found at: https://github.com/binfu0728/RASP-A-new-method-for-single-puncta-detection-in-complex-cellular-backgrounds (GitHub repository, MATLAB code). The version of pyRASP used in this paper may be found at: https://zenodo.org/doi/10.5281/zenodo.10688132 (pyRASP, version used in paper). The GitHub for pyRASP may be found at: https://github.com/TheLeeLab/pyRASP (GitHub repository for pyRASP). Raw data supporting Figure 5 may be found at https://doi.org/10.17867/10000195 (PDF)
RASP (ZIP)
pyRASP (ZIP)
