# Supplementary material for: RASP: Optimal Single Puncta Detection in Complex Cellular Backgrounds
Source: J Phys Chem B. 2024 Apr 9;128(15):3585–97. doi: 10.1021/acs.jpcb.4c00174 (PMC11033865; doi:10.1021/acs.jpcb.4c00174)
Supplement: Supplementary file 1 — jp4c00174_si_001.pdf [file jp4c00174_si_001.pdf]

Supplementary Information
RASP: Optimal Single Puncta Detection in Complex Cellular Backgrounds

Bin Fu,[†,‡] Emma E. Brock,[†,‡] Rebecca Andrews,[†,‡] Jonathan C. Breiter,[†,¶,‡] Ru Tian,[†,¶,‡] Christina E. Toomey,[§,‖,‡] Joanne Lachica,[§,#,‡] Tammaryn Lashley,[§,‖,‡] Mina Ryten,[@,‡] Nicholas W. Wood,[⊥,‡] Michele Vendruscolo,[¶,‡] Sonia Gandhi,[⊥,#,‡] Lucien E. Weiss,[△] Joseph S. Beckwith,[*,†,‡] and Steven F. Lee[*,†,‡]

†*Yusuf Hamied Department of Chemistry, Lensfield Road, Cambridge, CB2 1EW, UK*

‡*Aligning Science Across Parkinson's (ASAP) Collaborative Research Network, Chevy Chase, MD, 20815, USA*

¶*Centre for Misfolding Diseases, Yusuf Hamied Department of Chemistry, University of Cambridge, Cambridge, UK*

§*The Queen Square Brain Bank for Neurological Disorders, Department of Clinical and Movement Neuroscience, UCL Queen Square Institute of Neurology, London, UK*

‖*Department of Neurodegenerative diseases, UCL Queen Square Institute of Neurology, London, UK*

⊥*Department of Clinical and Movement Neurosciences, UCL Queen Square Institute of Neurology, London, UK*

#*The Francis Crick Institute, King's Cross, London, UK*

@*Great Ormond Street Institute of Child Health, University College London, London, UK*

△*Department of Engineering Physics, Polytechnique Montréal, Montréal, Québec, Canada*

E-mail: jsb92@cam.ac.uk; sl591@cam.ac.uk

Contents

S1 Precision and sensitivity for bead detection **S3**

S2 Validation of boundary selection method **S4**

S3 Precision and sensitivity on puncta detection on human brain tissue **S6**

S4 Gallery of puncta detected by RASP **S8**

S5 RASP with ThunderSTORM and PeakFit **S9**

S6 Rejecting out-of-focus images **S12**

S7 Diffraction-limited area threshold determination **S15**

S8 Colocalisation likelihood bootstrapping **S16**

S9 Intensity and background estimation validation **S17**

S10 RRID Table **S20**

S11 Patient Information Table **S21**

S12 Staining Plan Table **S22**

S13 Declarations **S23**

Supplementary Note S1: Precision and sensitivity for bead detection

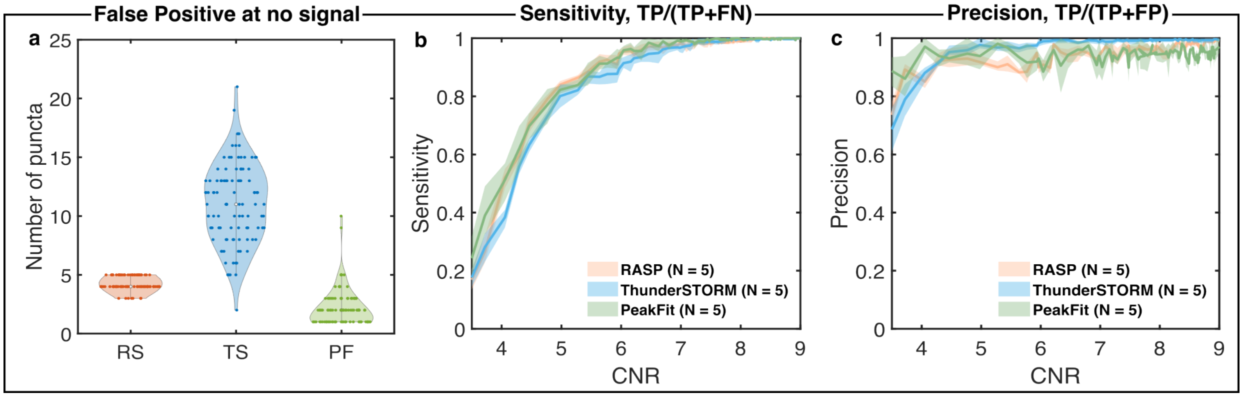

Figure S1: Precision and sensitivity curve for bead detection. a) Averaged number of locations detected from 5 different field-of-views at no signal (*i.e.* laser off) for RASP, ThunderSTORM, and PeakFit. **b)** Sensitivity comparison of RASP, ThunderSTORM, and PeakFit for 5 different field-of-views where the ground truth was determined from the highest CNR image. **c)** Precision comparison of RASP, ThunderSTORM, and PeakFit for 5 different field-of-views where the ground truth was determined from the highest CNR image.

In addition to the Jaccard index, we present additional metrics—sensitivity, precision, and the number of false positives in the absence of signal, to provide a more comprehensive evaluation of RASP's performance in sub-diffraction bead detection, in the absence of structured background. For the number of false positives in the absence of signal, Fig. S1**a**, the best performance would be as few false positives detected as possible. RASP, ThunderSTORM, and PeakFit detected 4.0 ± 0.46, 11.0 ± 3.37, and 2.21 ± 1.54 false positives per FoV respectively. RASP perfoms similarly to PeakFit in this case. For sensitivity (Fig. S1**b**), the curves from RASP, ThunderSTORM, and PeakFit overlap from low CNR to high CNR, indicating that the three codes have the same sensitivity. However, for precision (Fig. S1**c**), ThunderSTORM finds higher numbers false positives in the absence of signal, thus it performs less effectively compared to RASP and PeakFit at low CNR. Taken together, these show that RASP performs as well as state-of-the-art puncta detection codes in the case of no structured background.

Supplementary Note S2: Validation of boundary selection method

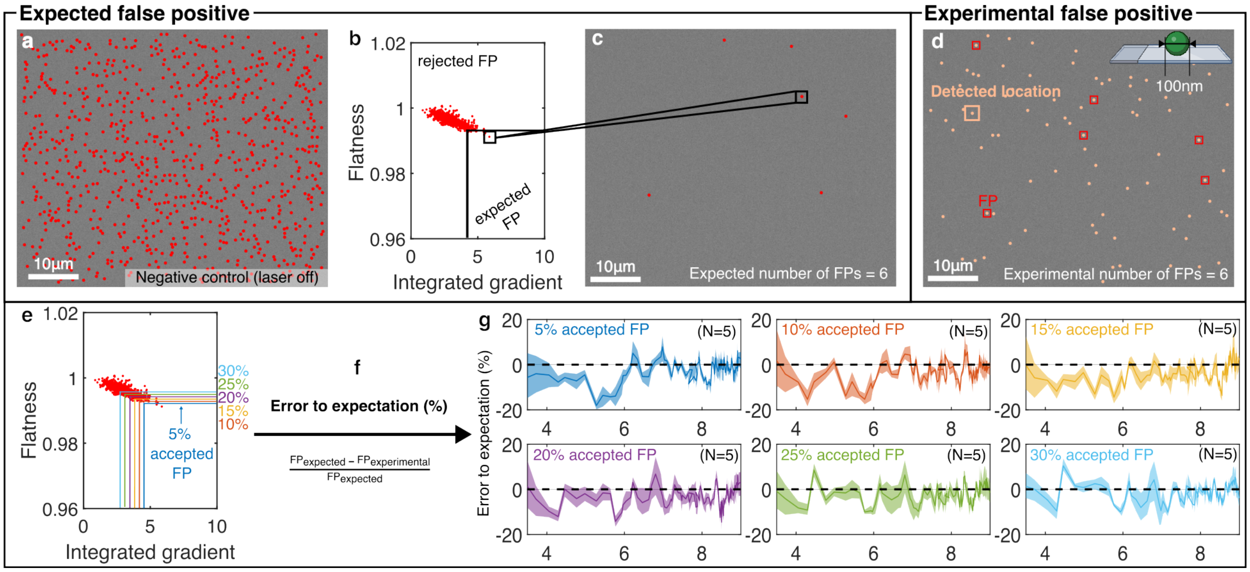

Figure S2: Validation for the boundary selection method. **a)** Detected locations in a negative control (*i.e.* background only) image from the sub-diffraction bead experiment. **b)** Decision boundary in the steepness and integrated dimension for all detected locations, accepting 5% false positives in each dimension separately. **c)** False positives remaining after implementing the decision boundary. 6 detected locations are left, which are recorded as the expected number of false positives (FPs) based on the current background and decision boundary. **d)** Detected locations with CNR = 5.5. A comparison with ground truth locations identifies 6 detected locations as false positives, recorded as the experimental number of FPs. **e)** The decision boundary of accepting 5% to 30% FP in two dimensions seperately. **f)** The equation for calculating the percentage error between experimental FPs and expected FPs. **g)** The percentage error between expected FPs and experimental FPs by changing the percentage of FP accepted in each dimension from 5% to 30%. Elements of this Figure were created with BioRender.com.

FPs in detection are mainly due to background (structured and unstructured). By comparing the FPs after applying the decision boundary on negative control images with those in single-bead or complex tissue images, we validate the reliability of the boundary selection method (*i.e.* the reliability of the sensitivity and precision predicted based on the decision boundary). Initially, the steepness and integrated gradient were computed for detected locations (Fig. S2**a**) within a negative control image, representing background-only conditions. A boundary, allowing 5% accepted FPs in each dimension, was imposed (Fig. S2**b**), and the remaining FPs were considered the expected background FPs (Fig. S2**c**). This boundary was then applied to sub-diffraction bead data within a CNR range of 3.5 to 9, the same the CNR range as in Fig. 2**i**. The number of experimental FPs was recorded, and the error was com-

puted as the percentage difference between the expected and experimental FPs, divided by the expected number of FPs (Fig. S2**e**). The sub-diffraction bead data underwent the same procedure but employing different decision boundaries allowing 10%, 15%, 20%, 25%, and 30% accepted FPs. Fig. S2**f** presents the resulting percentage error between the expected and experimental numbers of FPs with different decision boundaries used. This convergence toward zero as CNR increases shows the reliability of the precision and sensitivity anticipated from the decision boundary.

Supplementary Note S3: Precision and sensitivity on puncta detection on human brain tissue

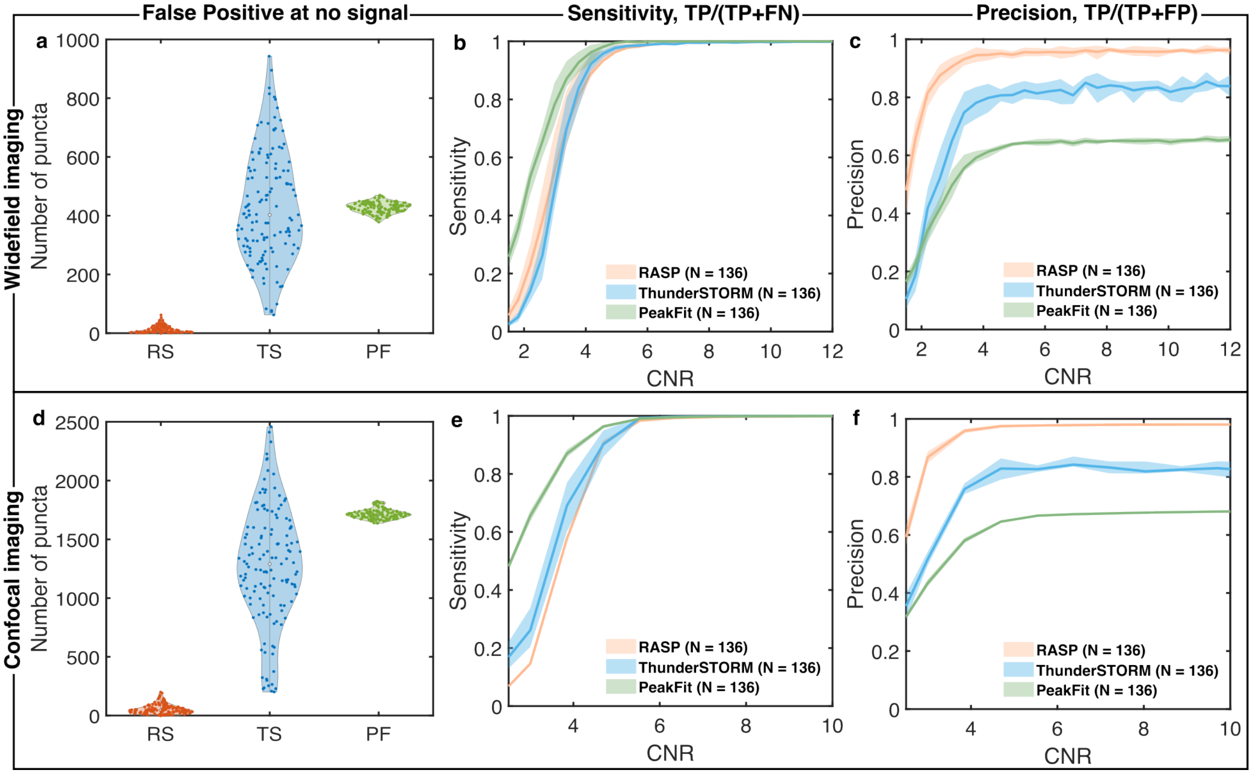

Figure S3: Precision and sensitivity curve for images from FFPE human brain slices. a) Averaged number of locations detected from 136 different field-of-views at no signal (*i.e.* only structured background from negative control) for RASP, ThunderSTORM, and PeakFit. **b)** The sensitivity comparison among RASP, ThunderSTORM, and PeakFit for 136 different field-of-views where the ground truth was determined from the simulated positions of puncta. **c)** The precision comparison among RASP, ThunderSTORM, and PeakFit for 136 different field-of-views where the ground truth was determined from the simulated positions of puncta.

In addition to the Jaccard index, we present additional metrics—sensitivity, precision, and the number of detected false positives on the negative control images, to provide a more comprehensive evaluation of RASP's performance in images containing structured background. The number of false positives detected in negative control images by RASP, ThunderSTORM, and PeakFit were 53 ± 42, 1278 ± 497, and 1716 ± 41 respectively for confocal imaging FoV (Fig. S3**a**) and 12 ± 12, 431 ± 184, and 427 ± 18 respectively for widefield imaging FoV (Fig. S3**d**). RASP detects significantly fewer false positives compared to PeakFit and ThunderSTORM. At high CNR (Fig. S3**b** and Fig. S3**e**), the sensitivity curves from RASP,

ThunderSTORM and PeakFit overlap, indicating these codes are equally sensitive. At high CNR (Fig. S3**c** and Fig. S3**f**), RASP (98.0% ± 0.6% for confocal and 95.9% ± 1.2% for widefield) outperforms ThunderSTORM (83.0% ± 3.9% for confocal and 83.8% ± 1.5% for widefield) and PeakFit (68.0% ± 0.4% for confocal and 65.1% ± 1.2% for widefield) in terms of precision. RASP thus effectively increases precision without sacrificing sensitivity.

Supplementary Note S4: Gallery of puncta detected by RASP

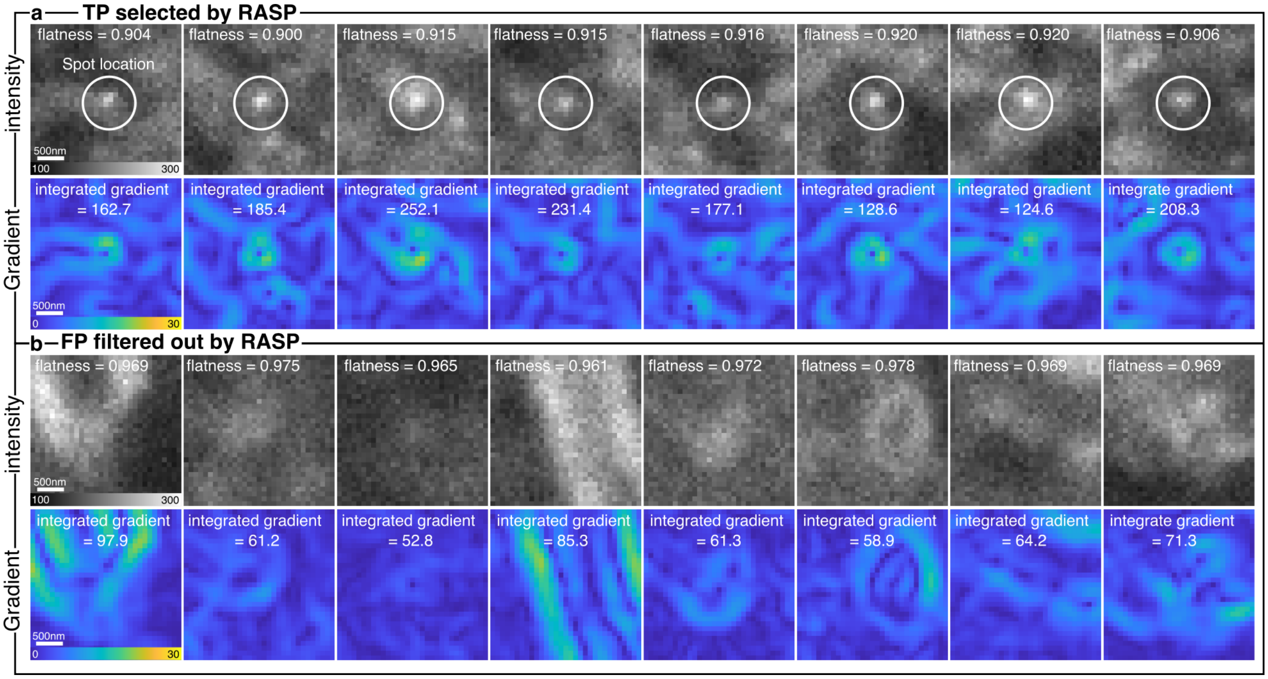

Figure S4: Gallery of true positive detections and false positive detections from RASP. a) The gallery of detected true positives (TPs) from RASP with flatness and integrated gradient. **b)** The gallery of removed false positives (FPs) from RASP with flatness and integrated gradient.

Supplementary Note S5: RASP with ThunderSTORM and PeakFit

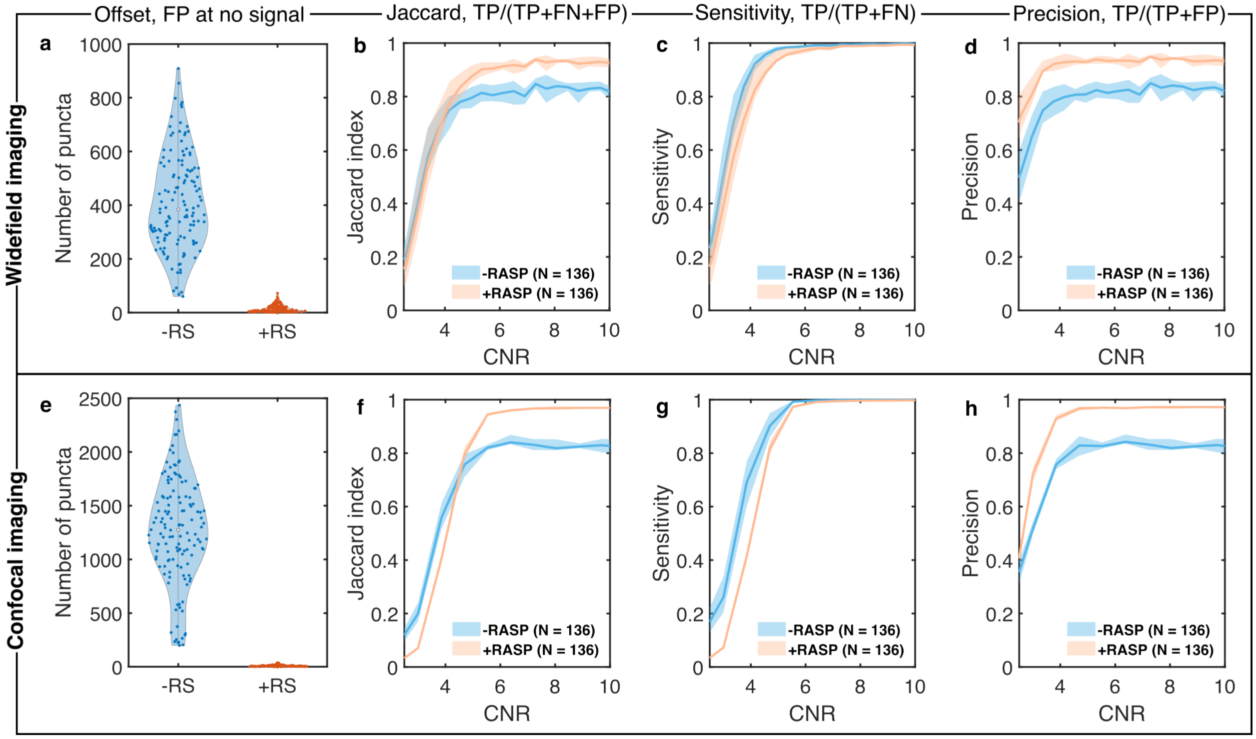

Figure S5: Increasing ThunderSTORM precision by applying RASP. a) and **e)** The number of puncta detected in 136 negative control images using ThunderSTORM with (+RS) and without (-RS) detected puncta being accepted or rejected using RASP's boundary filter in widefield and confocal imaging modes, respectively. **b)** and **f)** The Jaccard index for ThunderSTORM -RS and +RS for widefield imaging and confocal imaging, respectively. **c)** and **g)** The sensitivity for ThunderSTORM -RS and +RS for widefield imaging and confocal imaging, respectively. **d)** and **h)** The precision for ThunderSTORM -RS and +RS for widefield imaging and confocal imaging, respectively.

RASP, as a filtering technique for detected spots (Fig. 3), can be used to filter puncta detected by ThunderSTORM and PeakFit, thereby increasing their detection precision whilst maintaining their sensitivity. Initially, the flatness and integrated gradient values were calculated for all spots obtained through ThunderSTORM and PeakFit in widefield and confocal imaging from the dataset shown in Fig. 4. Then, the decision boundary established for RASP in Fig. 4 was applied directly to results from ThunderSTORM and PeakFit.

At high CNR, for ThunderSTORM, the filtering procedure results in an increase in the Jaccard index (90.2% ± 4.2% for widefield and 96.9% ± 0.6% for confocal) compared to the

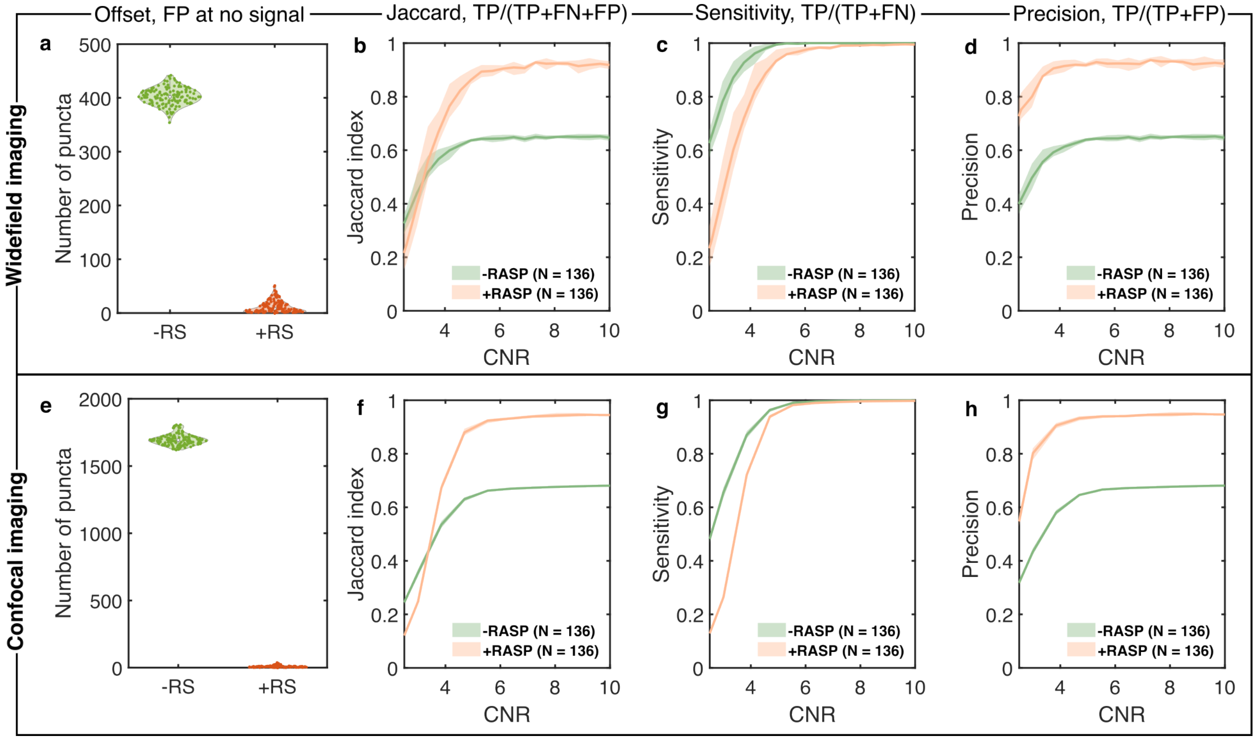

Figure S6: Increasing PeakFit precision by applying RASP. a) and **e)** The number of puncta detected in 136 negative control images using PeakFit with (+RS) and without (-RS) detected puncta being accepted or rejected using RASP's boundary filter in widefield and confocal imaging modes, respectively. **b)** and **f)** The Jaccard index for PeakFit -RS and +RS for widefield imaging and confocal imaging, respectively. **c)** and **g)** The sensitivity for PeakFit -RS and +RS for widefield imaging and confocal imaging, respectively. **d)** and **h)** The precision for PeakFit -RS and +RS for widefield imaging and confocal imaging, respectively.

original Jaccard index without RASP's filtering procedure (81.3% ± 4.5% for widefield and 83.4% ± 2.3% for confocal), Fig. S5**b** and **f**. The enhancement in the Jaccard index primarily arises from an increase in precision: 93.9% ± 2.0% for widefield and 97.1% ± 0.6% for confocal with RASP filtering, compared to 82.4% ± 4.3% for widefield and 83.5% ± 2.3% for confocal without RASP filtering (Fig. S5**d** and **h**).

Similar improvements were observed for PeakFit, where the filtered locations show a higher Jaccard index (90.5% ± 1.8% for widefield and 94.5% ± 0.8% for confocal) in comparison to the Jaccard index (64.4% ± 1.6% for widefield and 68.2% ± 0.4% for confocal) without filtering (Fig. S6**b** and **f**). The majority of the improvement observed in the Jaccard index was due to improved precision: 92.4% ± 2.3% for widefield and 68.3% ± 0.4% for confocal

with RASP filtering and 64.4% ± 1.6% for widefield and 94.6% ± 0.8% for confocal without RASP filtering (Fig. S5**d** and **h**).

Applying the decision boundary from RASP to both ThunderSTORM and PeakFit detected spots demonstrated minimal impact on sensitivity (Fig. S5**c** and **g** and Fig. S6**c** and **g**). This observation suggests that RASP effectively filters false positives without compromising sensitivity. Hence, this shows that RASP is capable of efficiently refining true positive spot detections whilst maintaining sensitivity, rendering it compatible with other single molecule detection methods and codes.

Supplementary Note S6: Rejecting out-of-focus images

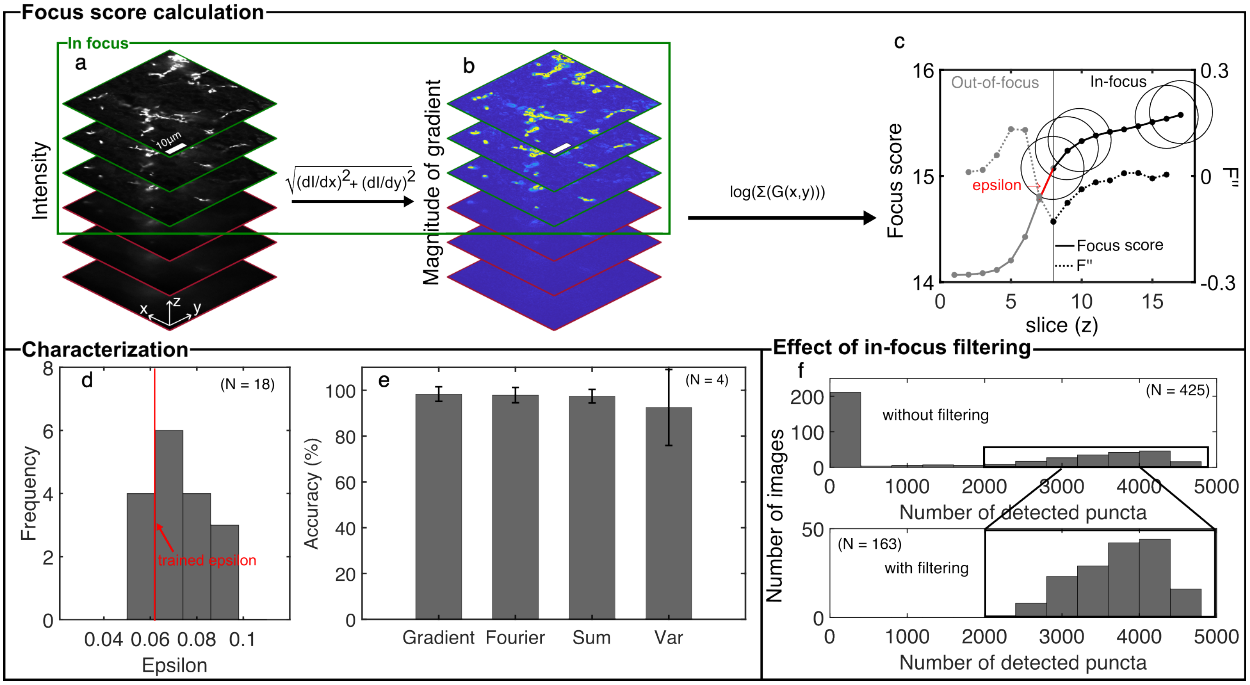

Figure S7: Image gradients efficiently select in-focus slices using DBSCAN. a). Intensity field of images within a Field of View (FoV). **b).** Gradient field derived from images, computed as the difference between the original image and a pixel-shifted version in both the x and y directions. **c).** Focus scores represented as the logarithm of the summed gradient field for each image within the chosen FoV, as well as the definition of the epsilon value. **d).** Distribution of epsilon values obtained from 18 FoVs. The threshold value was determined by the first quartile value of the distribution. **e).** Evaluation of in-focus filtering accuracy using various focus scoring metrics in comparison with human labelling. 25 FoVs were manually labelled by 4 different people. **f).** Application of in-focus filtering on 25 FoVs (17 images per FoV and 425 images in total) from 5 different imaging locations.

We have developed a strategy to distinguish between out-of-focus and in-focus images by introducing a focus score based on image gradient field to quantify blurriness. The gradient profile was determined by calculating the difference between the original image and a shifted version with a horizontal and vertical shift of 1 pixel (Fig. S7**b**). Subsequently, the focus score was computed as the logarithm of the summed gradient. Since different fields of view (FoVs) contain varying content, the absolute values of focus scores varied across different FoVs. Therefore, local classification, performed per FoV, proved to be more effective than global classification. Additionally, the image sharpness experiences the most significant change at the boundary between in-focus and out-of-focus states (Fig. S7**c**), which can be

effectively described by the difference in focus scores. This aligns with the concept of DBSCAN,[1] where the difference in focus scores can be used as the search radius for connected clusters (epsilon) (Fig. S7**c**). Therefore, a local DBSCAN procedure was applied to differentiate between in-focus and out-of-focus images within each specific FoV.

To determine the epsilon value, human annotated images of half in-focus FoVs were used as a starting point. Then, to reduce subjective standards for the half in-focus image among different people, the two images immediately preceding and following the labelled image were examined. Within these 5 images, the image with the minimum second derivative of the focus score was recorded as the transition image between in-focus and out-of-focus. Epsilons were determined from a set of 18 randomly selected FoVs, each captured from brain samples comprising 17 axial scans with a 500 nm step per axial scan. These samples were prepared as in Method Section B.1. Since epsilon represents the minimal separation distance between clusters, the first quartile value was selected, Fig. S7**d**.

The determined epsilon value was then applied to another 25 randomly selected FoVs in the same dataset. The results were subsequently compared against annotations from 4 different people for validation. Accuracy was assessed by calculating the ratio of true positives to the number of images per FoV. To account for the subjective standard from human annotations, we introduced a tolerance parameter, defined as the number of false positives accepted as true positives around the annotated transition image. The DBSCAN on the gradient scoring matrix performed the best with an accuracy of 98.4% $\pm$ 3.1% with a tolerance of 1 (Fig. S7**e**). Additionally, we evaluated alternative matrices[2] with their specific epsilon values. The focus scores based on the summed Fourier domain exhibited slightly less in accuracy (97.8% $\pm$ 3.3%). Also, the accuracy with tolerance = 1 from summed intensity is 97.4% $\pm$ 3.0%, variance is 92.5% $\pm$ 12.6%. The high accuracy with different scoring matrices also shows the effectiveness of DBSCAN in the in-focus image classification.

Furthermore, for the 25 FoVs used in the testing, we applied applied RASP to detect diffraction-limited puncta. The number of detected diffraction-limited puncta before (top) and after (bottom) in-focus filtering (Fig. S7**f**) showed that the results were affected by out-of-focus images, which our procedure can successfully reject.

Supplementary Note S7: Diffraction-limited area threshold determination

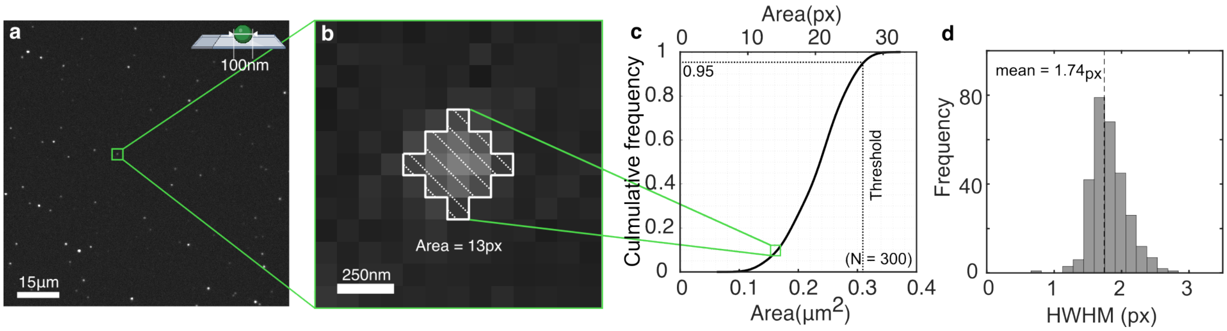

Figure S8: Calibration of the diffraction-limited puncta size on an image with 100 nm fluorescent beads. **a)** Images of 100 nm fluorescent beads were recorded with 100 ms exposure time (Method Section B.2) **b)** Zoom-in of a single bead. **c)** The cumulative density function of the detected area from the RASP with the same parameter used in the sub-diffraction beads experiment and the brain tissue experiment, with the threshold area value (27_{px}) for a diffraction limited object. **d)** The half-width-half-maximum (HWHM) of these beads from 2D Gaussian fits, showing that the mean rounded to the nearest integer is 2 pixels, elucidating its choice as the distance used in radiality calculations. Elements of this Figure were created using Biorender.com.

For post-filtering analysis, it is necessary to precisely and rapidly distinguish diffraction-limited puncta in an image from any puncta that are larger than the diffraction limit. Diffraction-limited puncta should be approximately identical, however various factors such as out-of-focus elements, optical aberrations, structured background, and other sources of noise (*e.g.* shot noise and read noise) introduce variability and cause different areas to be detected by RASP. In order to characterize the area associated with diffraction-limited puncta, we imaged 100 nm beads (Fig. S8**a**) from 5 different FoVs on Microscope 3 in Method Section A and analysed these images using RASP. Specifically, we used the same parameters from the sub-diffraction bead experiments shown in Fig. 2 and the brain tissue experiments shown in Fig. 4. The cumulative density function for detected spot area (Fig. S8**c**) was used to select a threshold (27_{px}) that includes 95% of all data. This threshold then was then applied to classify detected puncta into diffraction-limited and non-diffraction-limited categories.

Supplementary Note S8: Colocalisation likelihood bootstrapping

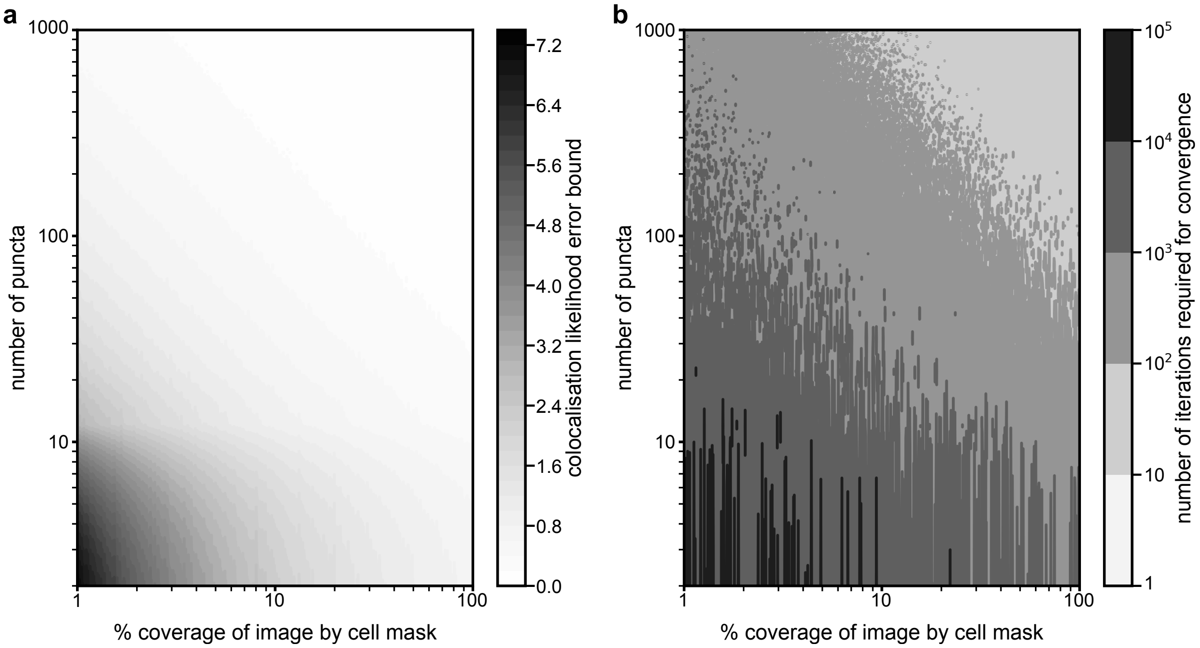

Figure S9: Colocalisation likelihood error calculations. a) The error bound on the colocalisation likelihood parameter as a function of % coverage of the image by cell mask, as well as number of puncta in the image. As expected, as the % coverage of the image by the cell mask and/or the number of puncta increases, the error bound decreases. **b)** Number of iterations needed for convergence of the CSR to be within 1% of the expected value (*i.e.* 1).

The colocalisation likelihood represents the probability of finding puncta inside a cell per FoV—above 1, it is more probable to find one inside the cell as compared to a uniform distribution. We also compute the error on this parameter using simulations of the same number of uniformly distributed puncta (so called complete spatial randomness data, CSR). To assess the error bounds on the colocalisation likelihood of real data, we simulate N iterations until the mean converges to within 1% of the value expected from a uniform distribution of spots across the image (computed from the faction of image occupied by the cell mask). NB that the 1% value is a user-tunable parameter. The error bound of these CSR data are then used as the error bound on the real colocalisation likelihood. As can be seen from Fig. S9, these error bounds and number of iterations scale predictably with cell mask converage and number of spots per image—with a more well-covered image and more

spots (*i.e.* a more well-sampled distribution) the error is lessened.

Supplementary Note S9: Intensity and background estimation validation

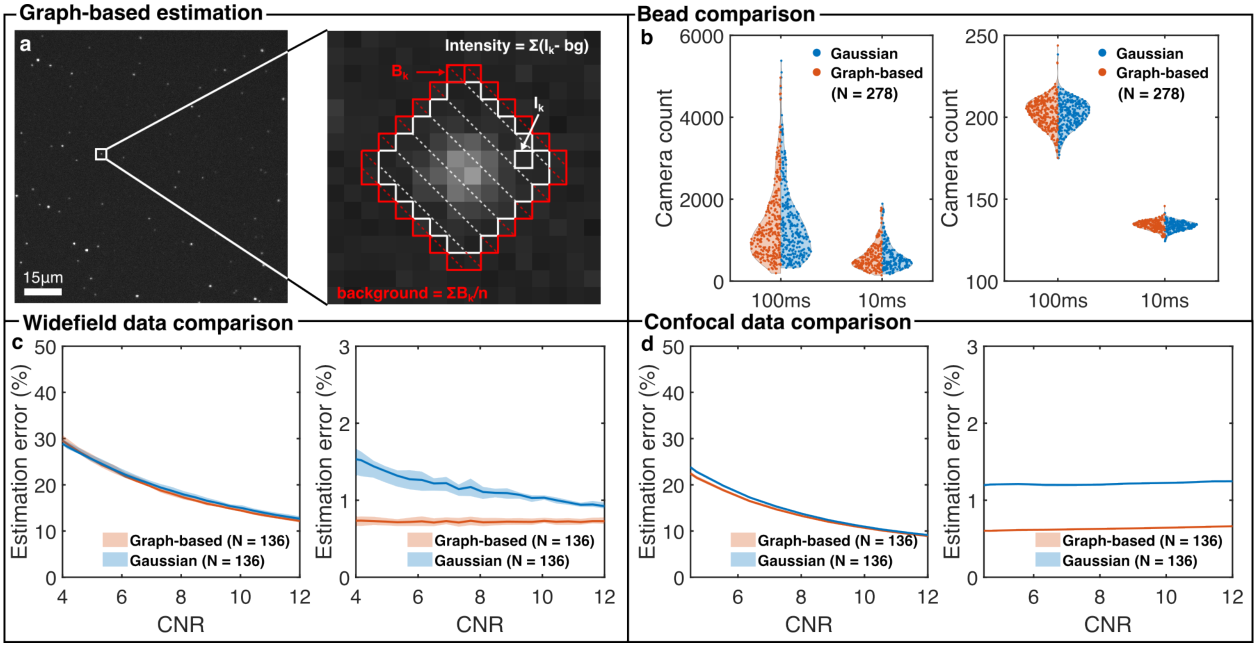

Figure S10: Graph-based intensity and background estimation for diffraction-limited puncta. a) An image of 100 nm fluorescent beads. Background is calculated by averaging the red pixels within the image. The intensity is calculated by summing white pixels after subtracting the estimated background value from each pixel. **b)** The comparison between using a symmetric 2D Gaussian function and nonlinear least squares fitting and the graph-based method for measuring intensity and background of sub-diffraction beads. Two different exposure time with the same laser power were used, 10 ms and 100 ms, to test the performance at different CNRs. **c)** Comparison between using 2D Gaussian fitting and the graph-based method to determine the intensity and background in images of FFPE brains taken using widefield imaging. **d)** Comparison between using 2D Gaussian fitting and the graph-based method to determine the intensity and background in images of FFPE brains taken using confocal imaging.

To accurately and efficiently determine the intensity and background of fluorescent puncta in a large dataset, we developed a graph-based method quantifying the intensity and the background from pixel values directly instead of fitting a 2D Gaussian model to the data. For the graph-based method, firstly, for each detected puncta from RASP, the centroid was recorded. Next, a disk-shaped binary mask with a radius of 5_{px} was generated (Fig. S10**a**). For the background calculation, the pixel values (B_{k}) that are 1_{px} away from the mask are averaged. Subsequently, for the intensity calculation, pixel values (I_{k}) in the disk-shaped

mask are summed after subtraction of the calculated background value using

$$\text{background} = \frac{1}{n}\sum_{\text{k}=1}^{n} B_{\text{k}} \tag{S1}$$

$$\text{I}_{\text{true}} = \sum_{k=1}^{n}(\text{I}_{\text{k}} - \text{background}). \tag{S2}$$

We firstly validated this method in a sub-diffraction bead experiment where the effect from the structured background is minimal. The 100 nm beads were prepared by the same protocol mentioned in Method Section B.2 but imaged on Microscope 3 with 561 nm laser. Two different exposure time with the same laser power (10 ms and 100 ms) were used for testing the accuracy of the graph-based method under low and high CNR case. The result from the graph-based estimation was compared with to fitting the detected spots with a symmetric 2D Gaussian function using nonlinear least squares fitting, a common method for estimating the intensity and background in single-molecule data. For the intensity estimation (Fig. S10**b**), the graph-based estimation yielded a slightly lower estimated value (1387 ± 899 for 100 mW and 605 ± 326 for 10 mW) compared to the 2D Gaussian fitting result (1410 ± 951 for 100 mW and for 610 ± 332 for 10 mW). For the background estimation (Fig. S10**b**), the two methods yielded similar distributions (Graph-based: 202 ± 8.9 for 100ṁW and 134 ± 2.8 for 10 mW, and 2D Gaussian: 201 ± 8.5 for 100ṁW and 133 ± 2.7 for 10 mW).

We then validated this method using images of FFPE antibody-stained human brain tissue, *i.e.* an image of a complex sample containing structured background. The data used are the same as the data in Fig. 4 where the simulated puncta and large features were added to real negative control brain images. The calculation of the ground truth intensity and the background is discussed in Method Section C. The estimated intensity and background values determined by graph-based method and the 2D Gaussian fitting were compared over the CNR range where RASP performs well. The error to the ground truth data was calculated

using

$$\text{error} = \frac{|\text{estimation} - \text{ground truth}|}{\text{ground truth}}. \tag{S3}$$

For the widefield data (Fig. S10**c**), the graph-based estimation (18.5% ± 5.0% on average) gave similar results to the 2D Gaussian fitting (18.9% ± 4.8% on average) in the intensity estimation. The error in background determination was low for both methods: 0.7% ± 0.01% on average for the graph-based estimation and 1.15% ± 0.17% on average for the 2D Gaussian fitting. Similar behaviour was found for the confocal data (Fig. S10**d**), with the graph-based estimation (8.4% ± 4.7% on average) giving similar results to the 2D Gaussian fitting (8.7% ± 5.0% on average) in the intensity estimation. Again, the error in the background determination was low for both methods: 0.71% ± 0.08% on average for the graph-based estimation and 1.28% ± 0.06% on average for the 2D Gaussian fitting. The graph-based method performs similarly to the 2D Gaussian fitting method in images with and without structured background. However, the time required for the graph-based method (9.5 ms per image with 1600 puncta) is far less compared to the 2D Gaussian fitting method (3.4 s per image with 1600 puncta) in MATLAB on a i9-11900K CPU and 80GB RAM PC, which makes this method superior for large datasets.

Supplementary Note S10: RRID Table

Table S1: RRID/CAS Table

Item	Catalog ID	Stock concentration	Dilution factor	Concentration used (mg/ml)	RRID/CAS
rabbit polyclonal to α-synuclein (phospho S129)	AB59264	1 mg/ml	1:200	0.005	AB_2270761
mouse monoclonal (psyn/81A) to α-synuclein (phospho S129)	AB184674	1 mg/ml	1:500	0.002	AB_2819037
Antibody, IBA1	Wako – 019-19741	1.1 mg/ml	1:1000	0.0011	AB_839504
Antibody, MAP2	ab254143	1 mg/ml	1:500	0.002	AB_2936822
Antibody, Alexafluor 568 goat anti-mouse – A11031	A11031	2 mg/ml	1:200	0.01	AB_144696
Antibody, Alexafluor 568 goat anti-rabbit – A11011	A11011	2 mg/ml	1:200	0.01	AB_143157
Antibody, Alexafluor 488 goat anti- rabbit – A11008	A11008	2 mg/ml	1:200	0.01	AB_143165
Antibody, Alexafluor 488 goat anti-mouse – A11001	A11001	2 mg/ml	1:200	0.01	AB_2534069
Methylated Spirit	IMS005	-	-	-	64-17-5
Xylene	XYL005	-	-	-	1330-20-7
Methanol	8222835000	-	-	-	67-56-1
Ethanol	E7148-500ML	-	-	-	64-17-5
Hydrogen Peroxide	23615.261	-	-	-	7722-84-1
PBS tablets	18912014	-	-	-	7647-14-5
VECTASHIELD+ PLUS Antifade Mounting Medium	H-1900-10	-	-	-	AB_2336789
Tri-sodium Citrate (for antigen retrieval)	27830.294	-	-	-	6132-04-03
Citric Acid (for antigen retrieval)	C/6200/53	-	-	-	5949-29-1
Sudan Black (Concentrate)	199664-25G	-	-	-	4197-25-5
Tetraspeck beads	T7279	-	-	-	-
Frame-seal slide chambers	SLF0201	-	-	-	-
Poly-L-lysine	P4707	-	-	-	25104-18-1
0.02 µm syringe filters	6809-1102	-	-	-	-
ImageJ	-	-	-	-	SCR_003070
MATLAB	-	-	-	-	SCR_001622
ThunderSTORM	-	-	-	-	SCR_016897
SlideBook	-	-	-	-	SCR_014423
GDSC SMLM	-	-	-	-	SCR_022717

Supplementary Note S11: Patient Information Table

Table S2: Patient Information Table. Brain Bank for all patients was Imperial, region was Cingulate Cortex in all cases and all patients had pathalogical diagnosis of Parkinson's Disease. [a]Post Moterm Interval.

Case	Age	Sex	Onset	Duration	PMI[a]	Confounding pathology	Braak score (α-syn)	Braak score (tau)	Thal score (amyloid beta)
PD0969	73	M	66	7	24	-	3	1	3
PD0822	81	M	64	17	22	-	4	2	-
PD0596	85	M	70	15	16	-	4	2	-

Supplementary Note S12: Staining Plan Table

Table S3: Staining Plan Table. All tissue was Formalin-Fixed Paraffin-Embedded (FFPE) and pressure-cooked before staining. Sudan Black was added to tissue post staining. For further details see Method section B.1.

Case	Primary antibody 1	Secondary antibody 1	Primary antibody 2	Secondary antibody 2	Sudan Black
PD0969	IBA1, Rabbit	Anti-Rabbit Alexa Fluor 488	Anti-phosphorated α-synuclein, Mouse	Anti-Mouse Alexa Fluor 561	+
PD0822	IBA1, Rabbit	Anti-Rabbit Alexa Fluor 488	Anti-phosphorated α-synuclein, Mouse	Anti-Mouse Alexa Fluor 561	+
PD0596	IBA1, Rabbit	Anti-Rabbit Alexa Fluor 488	Anti-phosphorated α-synuclein, Mouse	Anti-Mouse Alexa Fluor 561	+
PD0969	MAP2, Mouse	Anti-Mouse Alexa Fluor 488	Anti-phosphorated α-synuclein, Rabbit	Anti-Rabbit Alexa Fluor 561	+
PD0822	MAP2, Mouse	Anti-Mouse Alexa Fluor 488	Anti-phosphorated α-synuclein, Rabbit	Anti-Rabbit Alexa Fluor 561	+
PD0596	MAP2, Mouse	Anti-Mouse Alexa Fluor 488	Anti-phosphorated α-synuclein, Rabbit	Anti-Rabbit Alexa Fluor 561	+

Supplementary Note S13: Declarations

Ethical approval for the study was granted from the Local Research Ethics committee of the National Hospital for Neurology and Neurosurgery.

References

(1) Ester, M.; Kriegel, H.-P.; Sander, J.; Xu, X. A Density-Based Algorithm for Discovering Clusters in Large Spatial Databases with Noise. Proceedings of the Second International Conference on Knowledge Discovery and Data Mining. Portland, Oregon, 1996; pp 226–231.

(2) Sun, Y.; Duthaler, S.; Nelson, B. J. Autofocusing in Computer Microscopy: Selecting the Optimal Focus Algorithm. *Microsc. Res. Tech.* **2004**, *65*, 139–149.
