# Supplementary figures and images for: RASP: Optimal Single Puncta Detection in Complex Cellular Backgrounds
Source: J Phys Chem B. 2024 Apr 9;128(15):3585–97. doi: 10.1021/acs.jpcb.4c00174 (PMC11033865; doi:10.1021/acs.jpcb.4c00174)

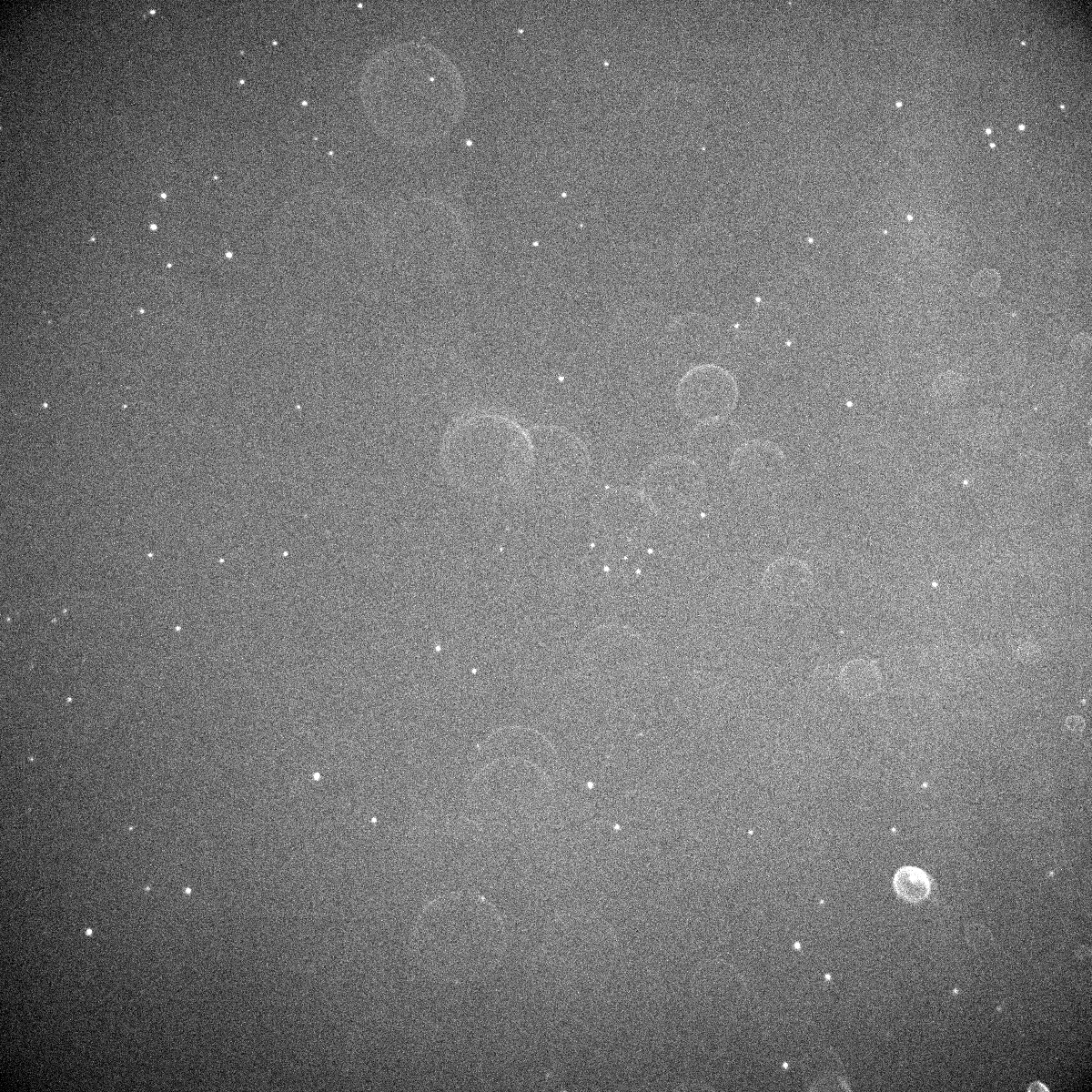

Supplement: Supplementary file 2 — jp4c00174_si_002.zip [file jp4c00174_si_002.zip › main_RASP/area_threshold/subbeads.tif]

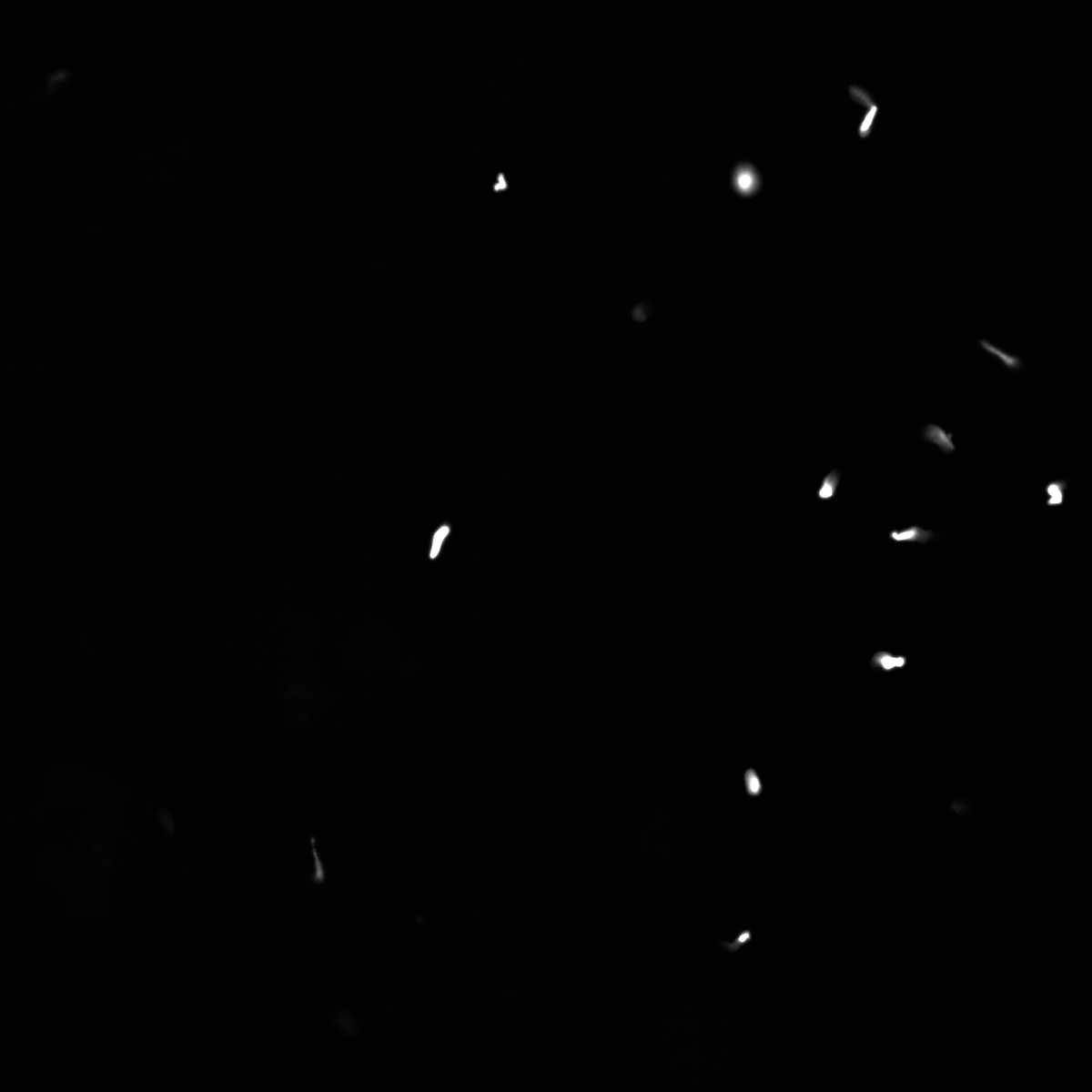

Supplement: Supplementary file 2 — jp4c00174_si_002.zip [file jp4c00174_si_002.zip › main_RASP/images/simulated_image.tif]

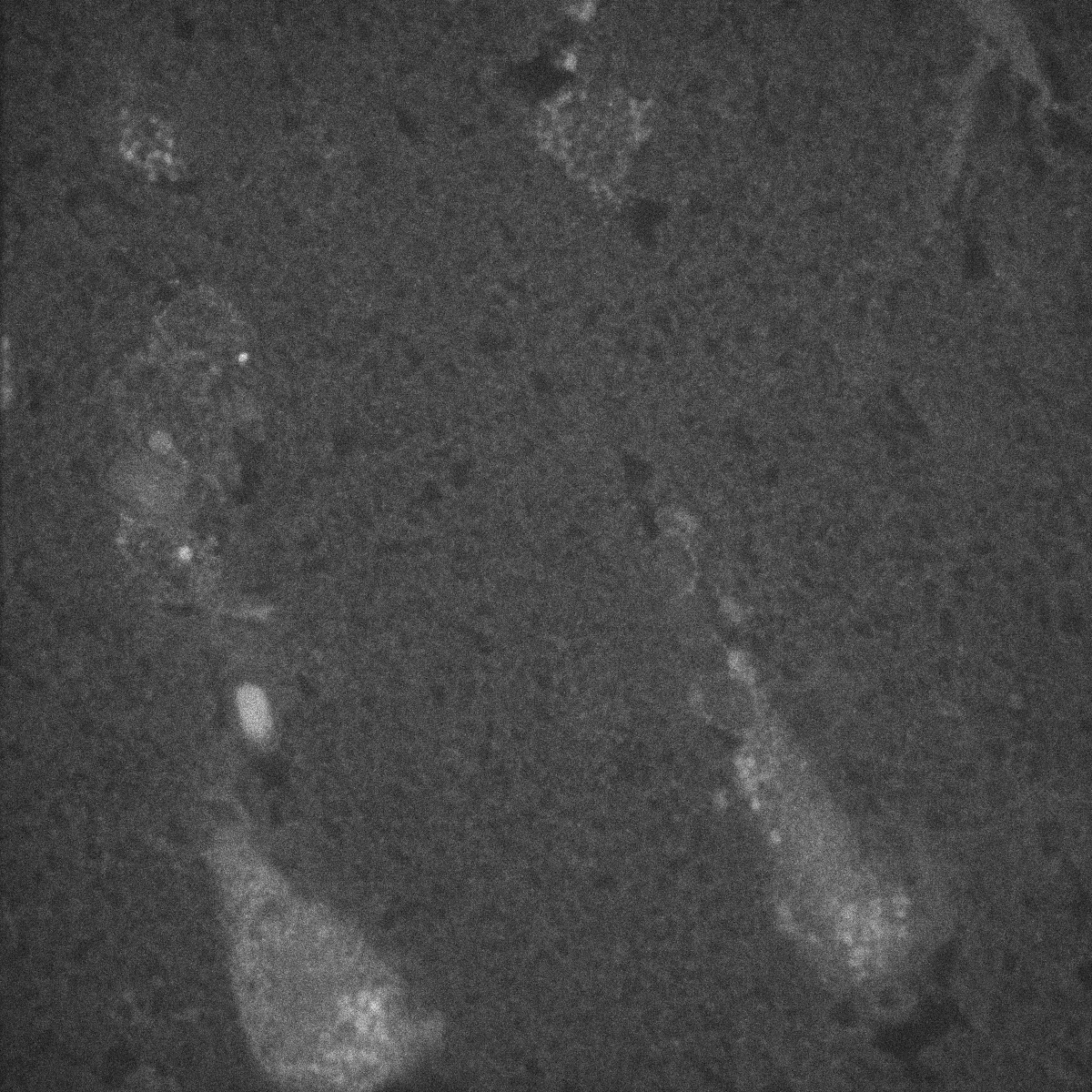

Supplement: Supplementary file 3 — jp4c00174_si_003.zip [file jp4c00174_si_003.zip › pyRASP_zip/negative_control/bg3.tif]

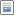

Supplement: Supplementary file 3 — jp4c00174_si_003.zip [file jp4c00174_si_003.zip › pyRASP_zip/docs/_build/html/_static/file.png]

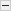

Supplement: Supplementary file 3 — jp4c00174_si_003.zip [file jp4c00174_si_003.zip › pyRASP_zip/docs/_build/html/_static/minus.png]

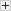

Supplement: Supplementary file 3 — jp4c00174_si_003.zip [file jp4c00174_si_003.zip › pyRASP_zip/docs/_build/html/_static/plus.png]

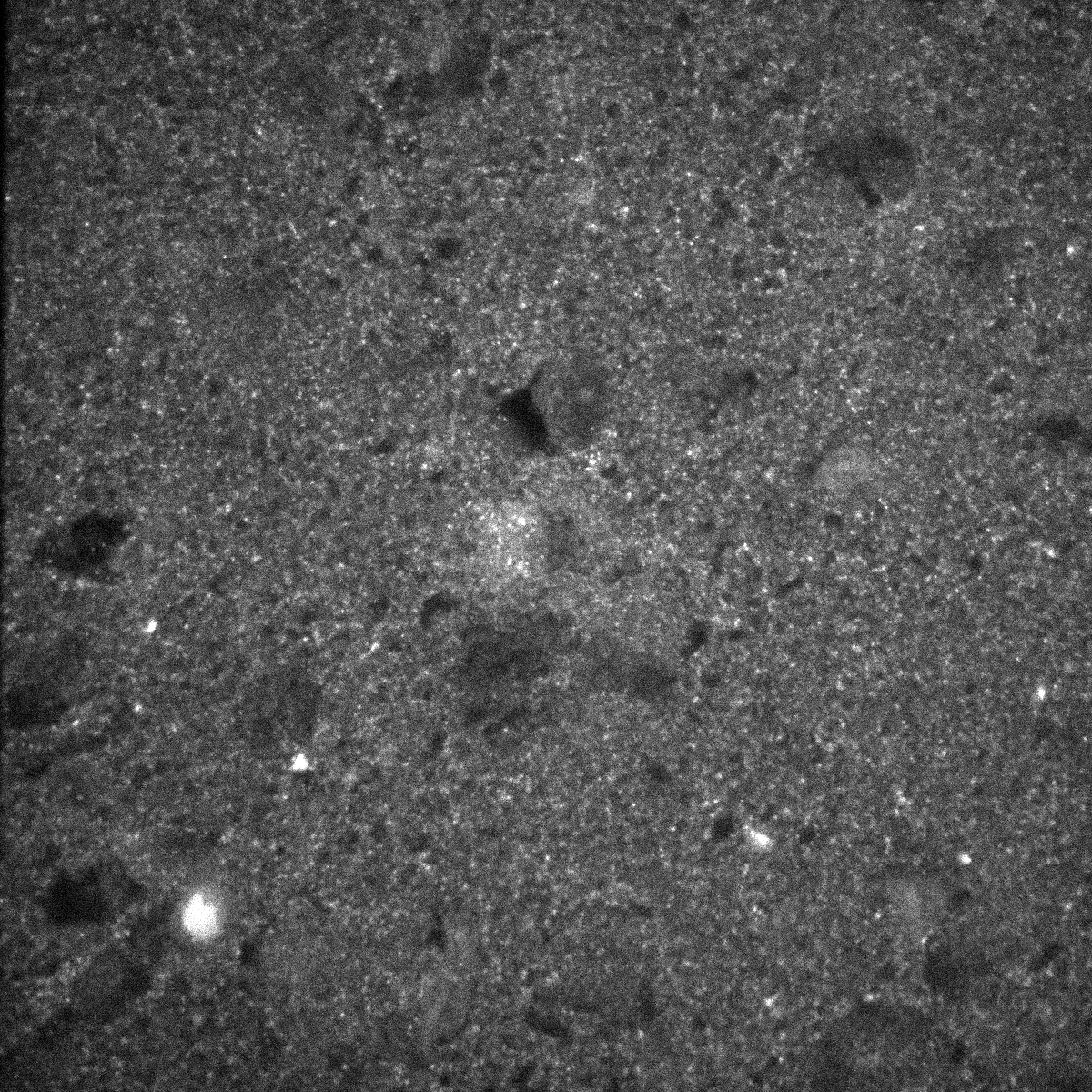

Supplement: Supplementary file 3 — jp4c00174_si_003.zip [file jp4c00174_si_003.zip › pyRASP_zip/example_images/Example_ProteinImage_C1.tif]

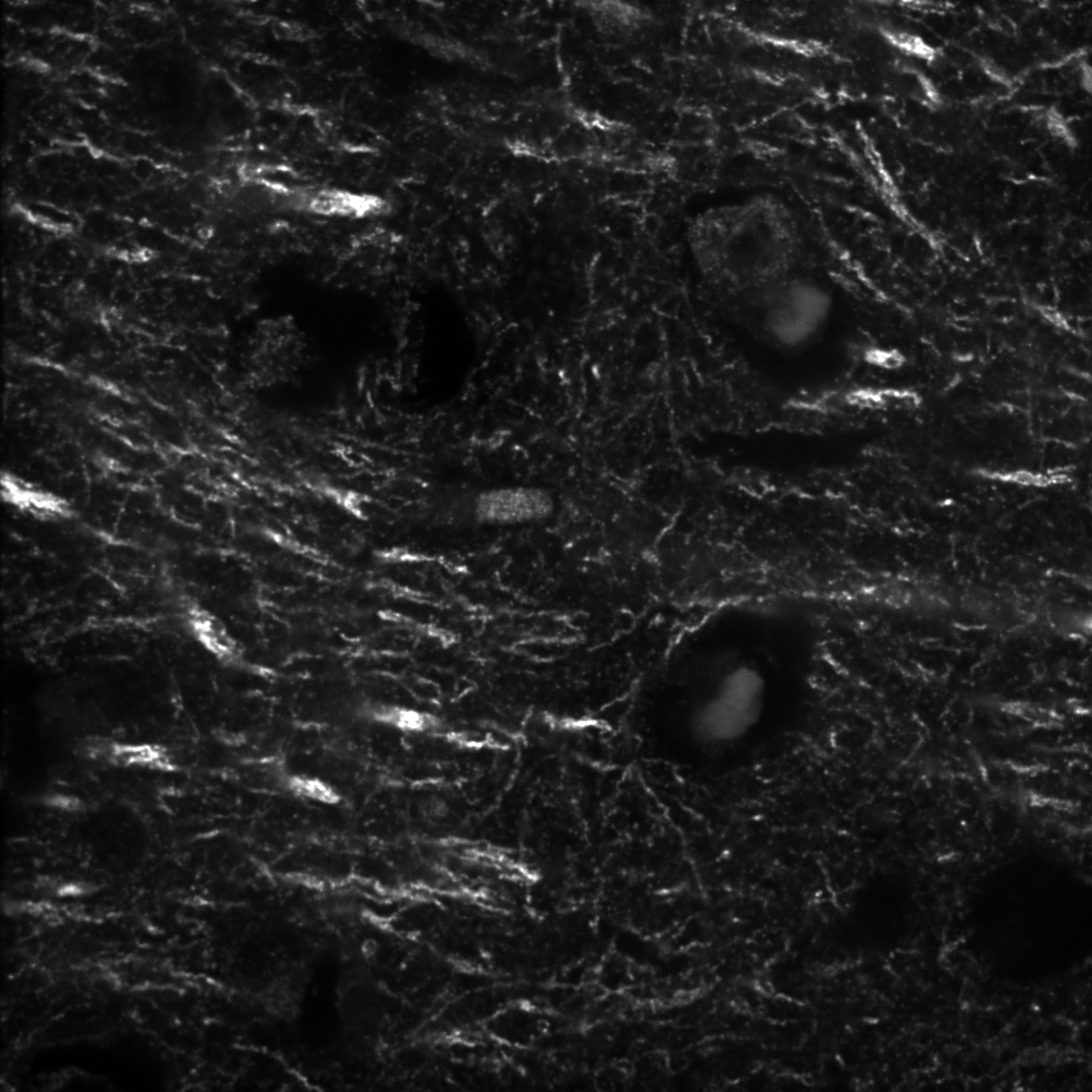

Supplement: Supplementary file 3 — jp4c00174_si_003.zip [file jp4c00174_si_003.zip › pyRASP_zip/example_images/Example_CellImage_C0.tif]
