# Supplementary material for: RASP: Optimal Single Puncta Detection in Complex Cellular Backgrounds
Source: J Phys Chem B. 2024 Apr 9;128(15):3585–97. doi: 10.1021/acs.jpcb.4c00174 (PMC11033865; doi:10.1021/acs.jpcb.4c00174)
Supplement: Supplementary file 3 — jp4c00174_si_003.zip [file jp4c00174_si_003.zip › pyRASP_zip/docs/_build/html/AnalysisFunctions.html]

AnalysisFunctions module — pyRASP v0.5.0 documentation


pyRASP

Contents:

- Introduction
- src
  - AnalysisFunctions module
    - `Analysis_Functions`
      - `Analysis_Functions.Gauss2DFitting()`
      - `Analysis_Functions.bincalculator()`
      - `Analysis_Functions.calculate_gradient_field()`
      - `Analysis_Functions.calculate_mask_fill()`
      - `Analysis_Functions.calculate_radiality()`
      - `Analysis_Functions.calculate_region_properties()`
      - `Analysis_Functions.calculate_spot_colocalisation_likelihood_ratio()`
      - `Analysis_Functions.compute_image_props()`
      - `Analysis_Functions.compute_spot_and_cell_props()`
      - `Analysis_Functions.compute_spot_props()`
      - `Analysis_Functions.create_filled_region()`
      - `Analysis_Functions.create_gaussian_kernel()`
      - `Analysis_Functions.create_kernel()`
      - `Analysis_Functions.default_spotanalysis_routine()`
      - `Analysis_Functions.detect_large_features()`
      - `Analysis_Functions.dilate_pixel()`
      - `Analysis_Functions.estimate_intensity()`
      - `Analysis_Functions.gen_CSRmats()`
      - `Analysis_Functions.generate_mask_and_spot_indices()`
      - `Analysis_Functions.infocus_indices()`
      - `Analysis_Functions.intensity_pixel_indices()`
      - `Analysis_Functions.make_datarray_cell()`
      - `Analysis_Functions.make_datarray_spot()`
      - `Analysis_Functions.rejectoutliers()`
      - `Analysis_Functions.ricker_wavelet()`
      - `Analysis_Functions.small_feature_kernel()`
      - `Analysis_Functions.test_spot_mask_overlap()`
  - IOFunctions module
  - PlottingFunctions module
  - RASPRoutines module

pyRASP

- src
- AnalysisFunctions module
- View page source

---

# AnalysisFunctions module

This class contains functions pertaining to analysis of images based on their
radiality, relating to the RASP concept.
jsb92, 2024/01/02

*class* AnalysisFunctions.Analysis\_Functions
:   Bases: `object`

    Gauss2DFitting(*image*, *pixel\_index\_list*, *expanded\_area=5*)
    :   Gets HWHM of PSFs from fitting Gaussians to spots (from pixel\_index\_list)
        Pixels expanded borders by n pixels (default 5)

        Parameters:
        :   - **image** (*array*) – image as numpy array
            - **pixel\_index\_list** (*list*) – list of pixel arrays
            - **area** (*expended*) – range to expand the pixel mask to

        Returns:
        :   **HWHMarray** (*array*) – array of half-width-half maxima from the fits

    bincalculator(*data*)
    :   bincalculator function
        reads in data and generates bins according to Freedman-Diaconis rule

        Parameters:
        :   **data** (*np.1darray*) – data to calculate bins

        Returns:
        :   **bins** (*np.1darray*) – bins for histogram according to Freedman-Diaconis rule

    calculate\_gradient\_field(*image*, *kernel*)
    :   Calculate the gradient field of an image and compute focus-related measures.

        Parameters:
        :   - **image** (*numpy.ndarray*) – The input image.
            - **kernel** (*numpy.ndarray*) – The kernel for low-pass filtering.

        Returns:
        :   - **filtered\_image** (*numpy.ndarray*) – Image after low-pass filtering.
            - **gradient\_x** (*numpy.ndarray*) – X-gradient of the filtered image.
            - **gradient\_y** (*numpy.ndarray*) – Y-gradient of the filtered image.
            - **focus\_score** (*numpy.ndarray*) – Focus score of the image.
            - **concentration\_factor** (*numpy.ndarray*) – Concentration factor of the image.

    calculate\_mask\_fill(*mask\_indices*, *image\_size*)
    :   calculate amount of image filled by mask.

        Parameters:
        :   - **mask\_indices** (*1D array*) – indices of pixels in mask
            - **image\_size** (*tuple*) – Image dimensions (height, width).

        Returns:
        :   **mask\_fill** (*float*) – proportion of image filled by mask.

    calculate\_radiality(*pil\_small*, *img*, *gradient\_x*, *gradient\_y*, *d=2*)
    :   Calculate radiality measures based on pixel neighborhoods and gradients.

        Parameters:
        :   - **pil\_small** (*list*) – List of pixel indices.
            - **img** (*numpy.ndarray*) – The input image.
            - **gradient\_x** (*numpy.ndarray*) – X-gradient of the image.
            - **gradient\_y** (*numpy.ndarray*) – Y-gradient of the image.
            - **d** (*integer*) – pixel ring size

        Returns:
        :   **radiality** (*numpy.ndarray*) – Radiality measures.

    calculate\_region\_properties(*binary\_mask*)
    :   Calculate properties for labeled regions in a binary mask.

        Parameters:
        :   **binary\_mask** (*numpy.ndarray*) – Binary mask of connected components.

        Returns:
        :   - **pixel\_index\_list** (*list*) – List containing pixel indices for each labeled object.
            - **areas** (*numpy.ndarray*) – Array containing areas of each labeled object.
            - **centroids** (*numpy.ndarray*) – Array containing centroids (x, y) of each labeled object.

    calculate\_spot\_colocalisation\_likelihood\_ratio(*spot\_indices*, *mask\_indices*, *image\_size*, *tol=0.01*, *n\_iter=100*)
    :   gets spot colocalisation likelihood ratio, as well as reporting error
        bounds on the likelihood ratio for one image

        Parameters:
        :   - **spot\_indices** (*1D array*) – indices of spots
            - **mask\_indices** (*1D array*) – indices of pixels in mask
            - **image\_size** (*tuple*) – Image dimensions (height, width).
            - **tol** (*float*) – default 0.01; tolerance for convergence
            - **n\_iter** (*int*) – default 100; number of iterations to start with

        Returns:
        :   - **colocalisation\_likelihood\_ratio** (*float*) – likelihood ratio of spots for mask
            - **perc\_std** (*float*) – standard deviation on this CLR based on bootstrapping
            - **meanCSR** (*float*) – mean of randomised spot data
            - **expected\_spots** (*float*) – number of spots we expect based on mask % of image
            - **n\_iter** (*int*) – how many iterations it took to converge

    compute\_image\_props(*image*, *k1*, *k2*, *thres=0.05*, *large\_thres=450.0*, *areathres=30.0*, *rdl=[50.0, 0.0, 0.0]*, *d=2*, *z\_planes=0*, *calib=False*)
    :   Gets basic image properties (dl\_mask, centroids, radiality)
        from a single image

        Parameters:
        :   - **image** (*array*) – image as numpy array
            - **k1** (*array*) – gaussian blur kernel
            - **k2** (*array*) – ricker wavelet kernel
            - **thres** (*float*) – percentage threshold
            - **areathres** (*float*) – area threshold
            - **rdl** (*array*) – radiality thresholds
            - **d** (*int*) – radiality ring
            - **z\_planes** (*array*) – If multiple z planes, give z planes
            - **calib** (*bool*) – If True, for radiality calibration

    compute\_spot\_and\_cell\_props(*image*, *image\_cell*, *k1*, *k2*, *prot\_thres=0.05*, *large\_prot\_thres=450.0*, *areathres=30.0*, *rdl=[50.0, 0.0, 0.0]*, *z=0*, *cell\_threshold1=200.0*, *cell\_threshold2=200*, *cell\_sigma1=2.0*, *cell\_sigma2=40.0*, *d=2*)
    :   Gets basic image properties (centroids, radiality)
        from a single image and compare to a cell mask from another image channel

        Parameters:
        :   - **image** (*array*) – image of protein stain as numpy array
            - **image** – image of cell stain as numpy array
            - **k1** (*array*) – gaussian blur kernel
            - **k2** (*array*) – ricker wavelet kernel
            - **prot\_thres** (*float*) – percentage threshold for protein
            - **large\_prot\_thres** (*float*) – Protein threshold intensity
            - **areathres** (*float*) – area threshold
            - **rdl** (*array*) – radiality thresholds
            - **z** (*array*) – z planes to image, default 0
            - **cell\_threshold1** (*float*) – 1st cell intensity threshold
            - **cell\_threshold2** (*float*) – 2nd cell intensity threshold
            - **cell\_sigma1** (*float*) – cell blur value 1
            - **cell\_sigma2** (*float*) – cell blur value 2
            - **d** (*integer*) – pixel radius value

    compute\_spot\_props(*image*, *k1*, *k2*, *thres=0.05*, *large\_thres=450.0*, *areathres=30.0*, *rdl=[50.0, 0.0, 0.0]*, *z=0*, *d=2*)
    :   Gets basic image properties (centroids, radiality)
        from a single image

        Parameters:
        :   - **image** (*array*) – image as numpy array
            - **k1** (*array*) – gaussian blur kernel
            - **k2** (*array*) – ricker wavelet kernel
            - **thres** (*float*) – percentage threshold
            - **areathres** (*float*) – area threshold
            - **rdl** (*array*) – radiality thresholds
            - **z** (*array*) – z planes to image, default 0
            - **d** (*int*) – Pixel radius value

    create\_filled\_region(*image\_size*, *indices\_to\_keep*)
    :   Fill a region in a boolean matrix based on specified indices.

        Parameters:
        :   - **image\_size** (*tuple*) – Size of the boolean matrix.
            - **indices\_to\_keep** (*list*) – List of indices to set as True.

        Returns:
        :   **boolean\_matrix** (*numpy.ndarray*) – Boolean matrix with specified indices set to True.

    create\_gaussian\_kernel(*sigmas*, *size*)
    :   Create a 2D Gaussian kernel.

        Parameters:
        :   - **sigmas** (*tuple*) – Standard deviations in X and Y directions.
            - **size** (*tuple*) – Size of the kernel.

        Returns:
        :   **kernel** (*numpy.ndarray*) – 2D Gaussian kernel.

    create\_kernel(*background\_sigma*, *wavelet\_sigma*)
    :   Create Gaussian and Ricker wavelet kernels.

        Parameters:
        :   - **background\_sigma** (*float*) – Standard deviation for Gaussian kernel.
            - **wavelet\_sigma** (*float*) – Standard deviation for Ricker wavelet.

        Returns:
        :   - **gaussian\_kernel** (*numpy.ndarray*) – Gaussian kernel for background suppression.
            - **ricker\_kernel** (*numpy.ndarray*) – Ricker wavelet for feature enhancement.

    default\_spotanalysis\_routine(*image*, *k1*, *k2*, *thres=0.05*, *large\_thres=450.0*, *areathres=30.0*, *rdl=[50.0, 0.0, 0.0]*, *d=2*)
    :   Daisy-chains analyses to get
        basic image properties (centroids, radiality)
        from a single image

        Parameters:
        :   - **image** (*array*) – image as numpy array
            - **k1** (*array*) – gaussian blur kernel
            - **k2** (*array*) – ricker wavelet kernel
            - **thres** (*float*) – percentage threshold
            - **areathres** (*float*) – area threshold
            - **rdl** (*array*) – radiality thresholds

        Returns:
        :   - **centroids** (*2D array*) – centroid positions per oligomer
            - **estimated\_intensity** (*numpy.ndarray*) – Estimated sum intensity per oligomer.
            - **estimated\_background** (*numpy.ndarray*) – Estimated mean background per oligomer.

    detect\_large\_features(*image*, *threshold1*, *threshold2=0*, *sigma1=2.0*, *sigma2=60.0*)
    :   Detects large features in an image based on a given threshold.

        Parameters:
        :   - **image** (*numpy.ndarray*) – Original image.
            - **threshold1** (*float*) – Threshold for determining features. Only this is
              used for the determination of large protein aggregates.
            - **threshold2** (*float*) – Threshold for determining cell features. If above
              0, gets used and cellular features are detected.
            - **sigma1** (*float*) – first gaussian blur width
            - **sigma2** (*float*) – second gaussian blur width

        Returns:
        :   **large\_mask** (*numpy.ndarray*) – Binary mask for the large features.

    dilate\_pixel(*index*, *image\_size*, *width=5*, *edge=1*)
    :   Dilate a pixel index to form a neighborhood.

        Parameters:
        :   - **index** (*int*) – Pixel index.
            - **image\_size** (*tuple*) – Image dimensions (height, width).
            - **width** – width of dilation (default 5)
            - **edge** – edge of dilation (default 1)

        Returns:
        :   **dilated\_indices** (*numpy.ndarray*) – Dilated pixel indices forming a neighborhood.

    estimate\_intensity(*image*, *centroids*)
    :   Estimate intensity values for each centroid in the image.

        Parameters:
        :   - **image** (*numpy.ndarray*) – Input image.
            - **centroids** (*numpy.ndarray*) – Centroid locations.

        Returns:
        :   - **estimated\_intensity** (*numpy.ndarray*) – Estimated sum intensity per oligomer.
            - **estimated\_background** (*numpy.ndarray*) – Estimated mean background per oligomer.

    gen\_CSRmats(*image\_z\_shape*)
    :   Generates empty matrices for the CSR

        Parameters:
        :   **image\_z\_shape** (*int*) – shape of new array

        Returns:
        :   - **clr** (*ndarray*) – empty array
            - **norm\_std** (*ndarray*) – empty array
            - **norm\_CSR** (*ndarray*) – empty array
            - **expected\_spots** (*ndarray*) – empty array
            - **n\_iter** (*ndarray*) – empty array

    generate\_mask\_and\_spot\_indices(*mask*, *centroids*, *image\_size*)
    :   makes mask and spot indices from xy coordinates

        Parameters:
        :   - **mask** (*2D array*) – boolean matrix
            - **centroids** (*2D array*) – xy centroid coordinates
            - **image\_size** (*tuple*) – Image dimensions (height, width).

        Returns:
        :   - **mask\_indices** (*1D array*) – indices of mask
            - **spot\_indices** (*1D array*) – indices of spots

    infocus\_indices(*focus\_scores*, *threshold\_differential*)
    :   Identify in-focus indices based on focus scores and a threshold differential.

        Parameters:
        :   - **focus\_scores** (*numpy.ndarray*) – Focus scores for different slices.
            - **threshold\_differential** (*float*) – Threshold for differential focus scores.

        Returns:
        :   **in\_focus\_indices** (*list*) – List containing the first and last in-focus indices.

    intensity\_pixel\_indices(*centroid\_loc*, *image\_size*)
    :   Calculate pixel indices for inner and outer regions around the given index.

        Parameters:
        :   - **centroid\_loc** (*2D array*) – xy location of the pixel.
            - **image\_size** (*tuple*) – Size of the image.

        Returns:
        :   - **inner\_indices** (*numpy.ndarray*) – Pixel indices for the inner region.
            - **outer\_indices** (*numpy.ndarray*) – Pixel indices for the outer region.

    make\_datarray\_cell(*clr*, *norm\_std*, *norm\_CSR*, *expected\_spots*, *n\_iter*, *columns*, *z\_planes='none'*)
    :   makes a datarray in pandas for cell information

        Parameters:
        :   - **clr** (*ndarray*) – colocalisation likelihood ratios
            - **estimated\_intensity** (*ndarray*) – estimated intensities
            - **estimated\_background** (*ndarray*) – estimated backgrounds
            - **columns** (*list* *of* *strings*) – column labels
            - **zp** (*string* *or* *int*) – if int, gives out z-plane version of datarray
            - **z\_planes** – z\_planes to put in array (if needed)

        Returns:
        :   **to\_save** (*pandas DataArray*) – pandas array to save

    make\_datarray\_spot(*centroids*, *estimated\_intensity*, *estimated\_background*, *columns*, *z\_planes=0*)
    :   makes a datarray in pandas for spot information

        Parameters:
        :   - **centroids** (*ndarray*) – centroid positions
            - **estimated\_intensity** (*ndarray*) – estimated intensities
            - **estimated\_background** (*ndarray*) – estimated backgrounds
            - **columns** (*list* *of* *strings*) – column labels
            - **z\_planes** – z\_planes to put in array (if needed); if int, assumes only
              one z-plane

        Returns:
        :   **to\_save** (*pandas DataArray*)

    rejectoutliers(*data*)
    :   rejectoutliers function
        # rejects outliers from data, does iqr method (i.e. anything below
        lower quartile (25 percent) or above upper quartile (75 percent)
        is rejected)

        Parameters:
        :   **data** (*np.1darray*) – data matrix

        Returns:
        :   **newdata** (*np.1darray*) – data matrix

    ricker\_wavelet(*sigma*)
    :   Create a 2D Ricker wavelet.

        Parameters:
        :   **sigma** (*float*) – Standard deviation for the wavelet.

        Returns:
        :   **wavelet** (*numpy.ndarray*) – 2D Ricker wavelet.

    small\_feature\_kernel(*img*, *large\_mask*, *img2*, *Gx*, *Gy*, *k2*, *thres*, *area\_thres*, *rdl*, *d=2*)
    :   Find small features in an image and determine diffraction-limited (dl) and non-diffraction-limited (ndl) features.

        Parameters:
        :   - **img** (*numpy.ndarray*) – Original image.
            - **large\_mask** (*numpy.ndarray*) – Binary mask for large features.
            - **img2** (*numpy.ndarray*) – Smoothed image for background suppression.
            - **Gx** (*numpy.ndarray*) – Gradient image in x-direction.
            - **Gy** (*numpy.ndarray*) – Gradient image in y-direction.
            - **k2** (*numpy.ndarray*) – The kernel for blob feature enhancement.
            - **thres** (*float*) – Converting real-valued image into a binary mask.
            - **area\_thres** (*float*) – The maximum area in pixels a diffraction-limited object can be.
            - **rdl** (*list*) – Radiality threshold [min\_radiality, max\_radiality, area].
            - **d** (*integer*) – pixel radius

        Returns:
        :   - **dl\_mask** (*numpy.ndarray*) – Binary mask for diffraction-limited (dl) features.
            - **centroids** (*numpy.ndarray*) – Centroids for dl features.
            - **radiality** (*numpy.ndarray*) – Radiality value for all features (before the filtering based on the radiality).
            - **idxs** (*numpy.ndarray*) – Indices for objects that satisfy the decision boundary.

    test\_spot\_mask\_overlap(*spot\_indices*, *mask\_indices*)
    :   Tests which spots overlap with a given mask.

        Parameters:
        :   - **spot\_indices** (*1D array*) – indices of spots
            - **mask\_indices** (*1D array*) – indices of pixels in mask

        Returns:
        :   **n\_spots\_in\_mask** (*float*) – number of spots that overlap with the mask.

Previous
Next

---

© Copyright 2024, Joseph S. Beckwith, Bin Fu, Steven F. Lee.

Built with Sphinx using a
theme
provided by Read the Docs.
