# Supplementary material for: RASP: Optimal Single Puncta Detection in Complex Cellular Backgrounds
Source: J Phys Chem B. 2024 Apr 9;128(15):3585–97. doi: 10.1021/acs.jpcb.4c00174 (PMC11033865; doi:10.1021/acs.jpcb.4c00174)
Supplement: Supplementary file 3 — jp4c00174_si_003.zip [file jp4c00174_si_003.zip › pyRASP_zip/docs/_build/html/IOFunctions.html]

IOFunctions module — pyRASP v0.5.0 documentation


pyRASP

Contents:

- Introduction
- src
  - AnalysisFunctions module
  - IOFunctions module
    - `IO_Functions`
      - `IO_Functions.load_json()`
      - `IO_Functions.make_directory()`
      - `IO_Functions.read_tiff()`
      - `IO_Functions.read_tiff_tophotons()`
      - `IO_Functions.save_analysis_params()`
      - `IO_Functions.save_as_json()`
      - `IO_Functions.write_tiff()`
  - PlottingFunctions module
  - RASPRoutines module

pyRASP

- src
- IOFunctions module
- View page source

---

# IOFunctions module

This class contains functions pertaining to IO of files based for the RASP code.
jsb92, 2024/01/02

*class* IOFunctions.IO\_Functions
:   Bases: `object`

    load\_json(*filename*)
    :   Loads data from a JSON file.

        Parameters:
        :   **filename** (*str*) – The name of the JSON file to load.

        Returns:
        :   **data** (*dict*) – The loaded JSON data.

    make\_directory(*directory\_path*)
    :   Creates a directory if it doesn’t exist.

        Parameters:
        :   **directory\_path** (*str*) – The path of the directory to be created.

    read\_tiff(*file\_path*)
    :   Read a TIFF file using the skimage library.

        Parameters:
        :   **file\_path** (*str*) – The path to the TIFF file to be read.

        Returns:
        :   **image** (*numpy.ndarray*) – The image data from the TIFF file.

    read\_tiff\_tophotons(*file\_path*, *QE=0.95*, *gain\_map=1.0*, *offset\_map=0.0*)
    :   Read a TIFF file using the skimage library.
        Use camera parameters to convert output to photons

        Parameters:
        :   - **file\_path** (*str*) – The path to the TIFF file to be read.
            - **QR** (*float*) – QE of camera
            - **gain\_map** (*matrix**, or* *float*) – gain map. Assumes units of ADU/photoelectrons
            - **offset\_map** (*matrix**, or* *float*) – offset map. Assumes units of ADU

        Returns:
        :   **image** (*numpy.ndarray*) – The image data from the TIFF file.

    save\_analysis\_params(*analysis\_p\_directory*, *to\_save*, *gain\_map=0*, *offset\_map=0*)
    :   saves analysis parameters.

        Parameters:
        :   - **analysis\_p\_directory** (*str*) – The folder to save to.
            - **to\_save** (*dict*) – dict to save of analysis parameters.
            - **gain\_map** (*array*) – gain\_map to save
            - **offset\_map** (*array*) – offset\_map to save

    save\_as\_json(*data*, *file\_name*)
    :   Saves data to a JSON file.

        Parameters:
        :   - **data** (*dict*) – The data to be saved in JSON format.
            - **file\_name** (*str*) – The name of the JSON file.

    write\_tiff(*volume*, *file\_path*, *bit=<class 'numpy.uint16'>*)
    :   Write a TIFF file using the skimage library.

        Parameters:
        :   - **volume** (*numpy.ndarray*) – The volume data to be saved as a TIFF file.
            - **file\_path** (*str*) – The path where the TIFF file will be saved.
            - **bit** (*int*) – Bit-depth for the saved TIFF file (default is 16).

        Notes

        The function uses skimage’s imsave to save the volume as a TIFF file.
        The plugin is set to ‘tifffile’ and photometric to ‘minisblack’.
        Additional metadata specifying the software as ‘Python’ is included.

Previous
Next

---

© Copyright 2024, Joseph S. Beckwith, Bin Fu, Steven F. Lee.

Built with Sphinx using a
theme
provided by Read the Docs.
