# Supplementary material for: RASP: Optimal Single Puncta Detection in Complex Cellular Backgrounds
Source: J Phys Chem B. 2024 Apr 9;128(15):3585–97. doi: 10.1021/acs.jpcb.4c00174 (PMC11033865; doi:10.1021/acs.jpcb.4c00174)
Supplement: Supplementary file 3 — jp4c00174_si_003.zip [file jp4c00174_si_003.zip › pyRASP_zip/docs/_build/html/PlottingFunctions.html]

PlottingFunctions module — pyRASP v0.5.0 documentation


pyRASP

Contents:

- Introduction
- src
  - AnalysisFunctions module
  - IOFunctions module
  - PlottingFunctions module
    - `Plotter`
      - `Plotter.histogram_plot()`
      - `Plotter.image_plot()`
      - `Plotter.image_scatter_plot()`
      - `Plotter.two_column_plot()`
  - RASPRoutines module

pyRASP

- src
- PlottingFunctions module
- View page source

---

# PlottingFunctions module

Class related to making figure-quality plots.

Probably best to set your default sans-serif font to Helvetica before you make
figures: https://fowlerlab.org/2019/01/03/changing-the-sans-serif-font-to-helvetica/

The maximum published width for a one-column
figure is 3.33 inches (240 pt). The maximum width for a two-column
figure is 6.69 inches (17 cm). The maximum depth of figures should
be 8 ¼ in. (21.1 cm).

panel labels are 8 point font, ticks are 7 point font,
annotations and legends are 6 point font.

*class* PlottingFunctions.Plotter(*poster=True*)
:   Bases: `object`

    histogram\_plot(*axs*, *data*, *bins*, *xlim=None*, *ylim=None*, *histcolor='gray'*, *xaxislabel='x axis'*, *alpha=1*, *histtype='bar'*, *density=True*)
    :   histogram\_plot function
        takes data and makes a histogram

        Parameters:
        :   - **data** (*np.1darray*) – data array
            - **bins** (*np.1darray*) – bin array
            - **xlim** (*boolean* *or* *list* *of* *two floats*) – default is None (which computes min/max of x), otherwise provide a min/max
            - **ylim** (*boolean* *or* *list* *of* *two floats*) – default is None (which computes min/max of y), otherwise provide a min/max
            - **histcolor** (*string*) – histogram colour (default is gray)
            - **xaxislabel** (*string*) – x axis label (default is ‘x axis’)
            - **alpha** (*float*) – histogram transparency (default 1)
            - **histtype** (*string*) – histogram type, default bar
            - **density** (*boolean*) – if to plot as pdf, default True

        Returns:
        :   **axs** (*axis*) – axis object

    image\_plot(*axs*, *data*, *vmin=None*, *vmax=None*, *cmap='gist\_gray'*, *cbar='on'*, *cbarlabel='photons'*, *label=''*, *labelcolor='white'*, *pixelsize=110*, *scalebarsize=5000*, *scalebarlabel='5$\\,\\mu$m'*, *alpha=1*, *plotmask=False*, *mask=None*)
    :   image\_plot function
        takes image data and makes an image plot

        Parameters:
        :   - **data** (*np.2darray*) – image
            - **vmin** (*float*) – minimum pixel intensity displayed (default 0.1%)
            - **vmax** (*float*) – minimum pixel intensity displayed (default 99.9%)
            - **cmap** (*string*) – colour map used; default gray)
            - **cbarlabel** (*string*) – colour bar label; default ‘photons’
            - **label** (*string*) – is any annotation
            - **labelcolor** (*string*) – annotation colour
            - **pixelsize** (*float*) – pixel size in nm for scalebar, default 110
            - **scalebarsize** (*float*) – scalebarsize in nm, default 5000
            - **scalebarlabel** (*string*) – scale bar label, default 5 um

        Returns:
        :   **axs** (*axis*) – axis object

    image\_scatter\_plot(*axs*, *data*, *xdata*, *ydata*, *vmin=None*, *vmax=None*, *cmap='gist\_gray'*, *cbar='on'*, *cbarlabel='photons'*, *label=''*, *labelcolor='white'*, *pixelsize=110*, *scalebarsize=5000*, *scalebarlabel='5$\\,\\mu$m'*, *alpha=1*, *scattercolor='red'*, *s=20*, *lws=0.75*)
    :   image\_plot function
        takes image data and makes an image plot

        Parameters:
        :   - **data** (*np.2darray*) – image
            - **xdata** (*np.1darray*) – scatter points, x
            - **ydata** (*np.1darray*) – scatter points, y
            - **vmin** (*float*) – minimum pixel intensity displayed (default 0.1%)
            - **vmax** (*float*) – minimum pixel intensity displayed (default 99.9%)
            - **cmap** (*string*) – colour map used; default gray)
            - **cbarlabel** (*string*) – colour bar label; default ‘photons’
            - **label** (*string*) – is any annotation
            - **labelcolor** (*string*) – annotation colour
            - **pixelsize** (*float*) – pixel size in nm for scalebar, default 110
            - **scalebarsize** (*float*) – scalebarsize in nm, default 5000
            - **scalebarlabel** (*string*) – scale bar label, default 5 um

        Returns:
        :   **axs** (*axis*) – axis object

    two\_column\_plot(*nrows=1*, *ncolumns=1*, *heightratio=[1]*, *widthratio=[1]*, *height=0*, *big=True*)
    :   two\_column\_plot function
        takes data and makes a two-column width figure

        Parameters:
        :   - **nrows** (*int*) – number of rows
            - **ncolumns** (*int*) – number of columns
            - **heightratio** (*list*) – list of heights of same length as nrows
            - **widthratio** (*list*) – list of widths of same length as ncolumns
            - **height** (*float*) – overridden height of figure
            - **big** (*boolean*) – if big is True, uses larger font sizes

        Returns:
        :   - **fig** (*figure*) – figure object
            - **ax** (*axes*) – axes object

Previous
Next

---

© Copyright 2024, Joseph S. Beckwith, Bin Fu, Steven F. Lee.

Built with Sphinx using a
theme
provided by Read the Docs.
