# Supplementary material for: RASP: Optimal Single Puncta Detection in Complex Cellular Backgrounds
Source: J Phys Chem B. 2024 Apr 9;128(15):3585–97. doi: 10.1021/acs.jpcb.4c00174 (PMC11033865; doi:10.1021/acs.jpcb.4c00174)
Supplement: Supplementary file 3 — jp4c00174_si_003.zip [file jp4c00174_si_003.zip › pyRASP_zip/docs/_build/html/RASPRoutines.html]

RASPRoutines module — pyRASP v0.5.0 documentation


pyRASP

Contents:

- Introduction
- src
  - AnalysisFunctions module
  - IOFunctions module
  - PlottingFunctions module
  - RASPRoutines module
    - `RASP_Routines`
      - `RASP_Routines.analyse_images()`
      - `RASP_Routines.analyse_round_images()`
      - `RASP_Routines.analyse_round_subfolder()`
      - `RASP_Routines.calibrate_area()`
      - `RASP_Routines.calibrate_radiality()`
      - `RASP_Routines.count_spots()`
      - `RASP_Routines.file_search()`
      - `RASP_Routines.get_infocus_planes()`
      - `RASP_Routines.save_analysis_results()`
      - `RASP_Routines.save_analysis_results_onesavefile()`
      - `RASP_Routines.single_image_analysis()`

pyRASP

- src
- RASPRoutines module
- View page source

---

# RASPRoutines module

This class contains functions that collect analysis routines for RASP.
jsb92, 2024/02/08

*class* RASPRoutines.RASP\_Routines(*defaultfolder=None*, *defaultarea=True*, *defaultd=True*, *defaultrad=True*, *defaultflat=True*, *defaultdfocus=True*, *defaultintfocus=True*, *defaultcellparams=True*, *defaultcameraparams=True*)
:   Bases: `object`

    analyse\_images(*folder*, *imtype='.tif'*, *thres=0.05*, *large\_thres=450.0*, *gsigma=1.4*, *rwave=2.0*, *oligomer\_string='C1'*, *cell\_string='C0'*, *if\_filter=True*, *im\_start=1*, *cell\_analysis=True*, *one\_savefile=False*, *disp=True*)
    :   analyses data from images in a specified folder,
        saves spots, locations, intensities and backgrounds in a folder created
        next to the folder analysed with \_analysis string attached
        also writes a folder with \_analysisparameters and saves analysis parameters
        used for particular experiment

        Parameters:
        :   - **folder** (*string*) – Folder containing images
            - **imtype** (*string*) – Type of images being analysed, default tif
            - **thres** (*float*) – fraction of bright pixels accepted
            - **large\_thres** (*float*) – large object intensity threshold
            - **gisgma** (*float*) – gaussian blurring parameter (default 1.4)
            - **rwave** (*float*) – Ricker wavelent sigma (default 2.)
            - **oligomer\_string** (*string*) – string for oligomer-containing data (default C1)
            - **string** (*cell*) – string for cell-containing data (default C0)
            - **if\_filter** (*boolean*) – Filter images for focus (default True)
            - **im\_start** (*integer*) – Images to start from (default 1)
            - **cell\_analysis** (*boolean*) – Parameter where script also analyses cell
              images and computes colocalisation likelihood ratios.
            - **one\_savefile** (*boolean*) – Parameter that, if true, doesn’t save a file
              per image but amalgamates them into one file
            - **disp** (*boolean*) – If true, prints when analysed an image stack.

    analyse\_round\_images(*folder*, *imtype='.tif'*, *thres=0.05*, *large\_thres=450.0*, *gsigma=1.4*, *rwave=2.0*, *oligomer\_string='C1'*, *cell\_string='C0'*, *if\_filter=True*, *im\_start=1*, *cell\_analysis=False*, *one\_savefile=True*)
    :   analyses data in a folder specified,
        folder has either “Round” in the title
        or multiple rounds below;
        structure as in Lee Lab Cambridge Experiment
        saves spots, locations, intensities and backgrounds in a folder created
        next to the folder analysed with \_analysis string attached
        also writes a folder with \_analysisparameters and saves analysis parameters
        used for particular experiment

        Parameters:
        :   - **folder** (*string*) – Folder containing images
            - **imtype** (*string*) – Type of images being analysed, default tif
            - **gisgma** (*float*) – gaussian blurring parameter (default 1.4)
            - **rwave** (*float*) – Ricker wavelent sigma (default 2.)
            - **oligomer\_string** (*string*) – string for oligomer-containing data (default C1)
            - **string** (*cell*) – string for cell-containing data (default C0)
            - **if\_filter** (*boolean*) – Filter images for focus (default True)
            - **im\_start** (*integer*) – Images to start from (default 1)
            - **one\_savefile** (*boolean*) – Parameter that, if true, doesn’t save a file
              per image but amalgamates them into one file
            - **cell\_analysis** (*boolean*) – Parameter where script also analyses cell
              images and computes colocalisation likelihood ratios.

    analyse\_round\_subfolder(*folder*, *k1*, *k2*, *rdl*, *imtype='.tif'*, *thres=0.05*, *large\_thres=450.0*, *gsigma=1.4*, *rwave=2.0*, *oligomer\_string='C1'*, *cell\_string='C0'*, *if\_filter=True*, *im\_start=1*, *cell\_analysis=False*, *one\_savefile=True*, *disp=True*)
    :   analyses data in a folder specified,
        folder has either “Round” in the title
        or multiple rounds below;
        structure as in Lee Lab Cambridge Experiment
        saves spots, locations, intensities and backgrounds in a folder created
        next to the folder analysed with \_analysis string attached
        also writes a folder with \_analysisparameters and saves analysis parameters
        used for particular experiment

        Parameters:
        :   - **folder** (*string*) – Folder containing images
            - **k1** (*matrix*) – convolution kernel 1
            - **k2** (*matrix*) – convolution kernel 2
            - **rdl** (*vector*) – radiality filter
            - **thres** (*float*) – fraction of bright pixels accepted
            - **large\_thres** (*float*) – large object intensity threshold
            - **imtype** (*string*) – Type of images being analysed, default tif
            - **gisgma** (*float*) – gaussian blurring parameter (default 1.4)
            - **rwave** (*float*) – Ricker wavelent sigma (default 2.)
            - **oligomer\_string** (*string*) – string for oligomer-containing data (default C1)
            - **string** (*cell*) – string for cell-containing data (default C0)
            - **if\_filter** (*boolean*) – Filter images for focus (default True)
            - **im\_start** (*integer*) – Images to start from (default 1)
            - **one\_savefile** (*boolean*) – Parameter that, if true, doesn’t save a file
              per image but amalgamates them into one file
            - **cell\_analysis** (*boolean*) – Parameter where script also analyses cell
              images and computes colocalisation likelihood ratios.
            - **disp** (*boolean*) – If True, outputs a message saying analysed image.

    calibrate\_area(*folder*, *imtype='.tif'*, *gsigma=1.4*, *rwave=2.0*, *large\_thres=10000.0*)
    :   Calibrates area threshold. Given a folder of bead images,
        analyses them and saves the radiality parameter to the .json file, as
        well as writing it to the current class radiality and flatness values

        Parameters:
        :   - **folder** (*string*) – Folder containing bead (bright) control tifs
            - **imtype** (*string*) – Type of images being analysed, default tif
            - **gisgma** (*float*) – gaussian blurring parameter (default 1.4)
            - **rwave** (*float*) – Ricker wavelent sigma (default 2.)

    calibrate\_radiality(*folder*, *imtype='.tif'*, *gsigma=1.4*, *rwave=2.0*, *accepted\_ratio=1*)
    :   Calibrates radility parameters. Given a folder of negative controls,
        analyses them and saves the radiality parameter to the .json file, as
        well as writing it to the current class radiality and flatness values

        Parameters:
        :   - **folder** (*string*) – Folder containing negative control tifs
            - **imtype** (*string*) – Type of images being analysed, default tif
            - **gsigma** (*float*) – gaussian blurring parameter (default 1.4)
            - **rwave** (*float*) – Ricker wavelent sigma (default 2.)
            - **accepted\_ratio** (*float*) – Percentage accepted of false positives

    count\_spots(*database*, *z\_planes*)
    :   Counts spots per z plane

        Parameters:
        :   - **database** (*pandas array*) – pandas array of spots
            - **z\_planes** (*np.1darray*) – is range of zplanes

        Returns:
        :   **n\_spots**

    file\_search(*folder*, *string1*, *string2*)
    :   Search for files containing ‘string1’ in their names within ‘folder’,
        and then filter the results to include only those containing ‘string2’.

        Parameters:
        :   - **folder** (*str*) – The directory to search for files.
            - **string1** (*str*) – The first string to search for in the filenames.
            - **string2** (*str*) – The second string to filter the filenames containing string1.

        Returns:
        :   **file\_list** (*list*) – A sorted list of file paths matching the search criteria.

    get\_infocus\_planes(*image*, *kernel*)
    :   Gets z planes that area in focus from an image stack

        Parameters:
        :   - **image** (*array*) – image as numpy array
            - **kernel** (*array*) – gaussian blur kernel

        Returns:
        :   **z\_planes** (*np.1darray*) – z\_plane range that is in focus

    save\_analysis\_results(*directory*, *file*, *to\_save*, *rsid*, *cell\_analysis=False*, *to\_save\_cell=0*, *cell\_mask=0*)
    :   Saves analysis results to the specified directory.

        Parameters:
        :   - **directory** (*str*) – The directory where the results will be saved.
            - **file\_path** (*str*) – The file path of the original data file.
            - **data\_to\_save** (*pandas.DataFrame*) – The data to be saved.
            - **rsid** (*float*) – The rsid value associated with the data.
            - **cell\_analysis** (*bool*) – Indicates whether cell analysis was performed (default False).
            - **cell\_data\_to\_save** (*pandas.DataFrame*) – The cell data to be saved (default None).
            - **cell\_mask** (*numpy.ndarray*) – The cell mask to be saved as a TIFF file (default None).

    save\_analysis\_results\_onesavefile(*analysis\_directory*, *file*, *to\_save*, *rsid*, *z\_planes*, *i*, *cell\_analysis=False*, *cell\_file=0*, *to\_save\_cell=0*, *cell\_mask=0*)
    :   Saves analysis results to the specified directory.

        Parameters:
        :   - **directory** (*str*) – The directory where the results will be saved.
            - **file\_path** (*str*) – The file path of the original data file.
            - **data\_to\_save** (*pandas.DataFrame*) – The data to be saved.
            - **rsid** (*float*) – The rsid value associated with the data.
            - **cell\_analysis** (*bool*) – Indicates whether cell analysis was performed (default False).
            - **cell\_data\_to\_save** (*pandas.DataFrame*) – The cell data to be saved (default None).
            - **cell\_mask** (*numpy.ndarray*) – The cell mask to be saved as a TIFF file (default None).

    single\_image\_analysis(*protein\_file*, *thres=0.05*, *large\_thres=450.0*, *gsigma=1.4*, *rwave=2.0*, *image\_size=200*, *save\_figure=False*, *cell\_analysis=False*, *cell\_file=None*)
    :   analyses data from specified image,
        presents spots, locations, intensities in a figure, with the option of
        saving this figure

        Parameters:
        :   - **file** (*string*) – image location
            - **thres** (*float*) – fraction of bright pixels accepted
            - **large\_thres** (*float*) – large object intensity threshold
            - **gisgma** (*float*) – gaussian blurring parameter (default 1.4)
            - **rwave** (*float*) – Ricker wavelent sigma (default 2.)
            - **image\_size** (*int*) – Amount of image to plot—by default plots 100x100
              chunk of an image to give you an idea, can scale up
            - **save\_figure** (*boolean*) – save the figure as an svg, default no
            - **cell\_analysis** (*boolean*) – Parameter where script also analyses cell
              images and computes colocalisation likelihood ratios.
            - **cell\_file** (*string*) – cell image location

Previous

---

© Copyright 2024, Joseph S. Beckwith, Bin Fu, Steven F. Lee.

Built with Sphinx using a
theme
provided by Read the Docs.
