# Supplementary material for: RASP: Optimal Single Puncta Detection in Complex Cellular Backgrounds
Source: J Phys Chem B. 2024 Apr 9;128(15):3585–97. doi: 10.1021/acs.jpcb.4c00174 (PMC11033865; doi:10.1021/acs.jpcb.4c00174)
Supplement: Supplementary file 3 — jp4c00174_si_003.zip [file jp4c00174_si_003.zip › pyRASP_zip/docs/_build/html/genindex.html]

Index — pyRASP v0.5.0 documentation


pyRASP

Contents:

- Introduction
- src

pyRASP

- Index

---

# Index

**A**
| **B**
| **C**
| **D**
| **E**
| **F**
| **G**
| **H**
| **I**
| **L**
| **M**
| **P**
| **R**
| **S**
| **T**
| **W**

## A

|  |  |
| --- | --- |
| - analyse\_images() (RASPRoutines.RASP\_Routines method) - analyse\_round\_images() (RASPRoutines.RASP\_Routines method) - analyse\_round\_subfolder() (RASPRoutines.RASP\_Routines method) | - Analysis\_Functions (class in AnalysisFunctions) - AnalysisFunctions   - module |

## B

|  |
| --- |
| - bincalculator() (AnalysisFunctions.Analysis\_Functions method) |

## C

|  |  |
| --- | --- |
| - calculate\_gradient\_field() (AnalysisFunctions.Analysis\_Functions method) - calculate\_mask\_fill() (AnalysisFunctions.Analysis\_Functions method) - calculate\_radiality() (AnalysisFunctions.Analysis\_Functions method) - calculate\_region\_properties() (AnalysisFunctions.Analysis\_Functions method) - calculate\_spot\_colocalisation\_likelihood\_ratio() (AnalysisFunctions.Analysis\_Functions method) - calibrate\_area() (RASPRoutines.RASP\_Routines method) - calibrate\_radiality() (RASPRoutines.RASP\_Routines method) | - compute\_image\_props() (AnalysisFunctions.Analysis\_Functions method) - compute\_spot\_and\_cell\_props() (AnalysisFunctions.Analysis\_Functions method) - compute\_spot\_props() (AnalysisFunctions.Analysis\_Functions method) - count\_spots() (RASPRoutines.RASP\_Routines method) - create\_filled\_region() (AnalysisFunctions.Analysis\_Functions method) - create\_gaussian\_kernel() (AnalysisFunctions.Analysis\_Functions method) - create\_kernel() (AnalysisFunctions.Analysis\_Functions method) |

## D

|  |  |
| --- | --- |
| - default\_spotanalysis\_routine() (AnalysisFunctions.Analysis\_Functions method) | - detect\_large\_features() (AnalysisFunctions.Analysis\_Functions method) - dilate\_pixel() (AnalysisFunctions.Analysis\_Functions method) |

## E

|  |
| --- |
| - estimate\_intensity() (AnalysisFunctions.Analysis\_Functions method) |

## F

|  |
| --- |
| - file\_search() (RASPRoutines.RASP\_Routines method) |

## G

|  |  |
| --- | --- |
| - Gauss2DFitting() (AnalysisFunctions.Analysis\_Functions method) - gen\_CSRmats() (AnalysisFunctions.Analysis\_Functions method) | - generate\_mask\_and\_spot\_indices() (AnalysisFunctions.Analysis\_Functions method) - get\_infocus\_planes() (RASPRoutines.RASP\_Routines method) |

## H

|  |
| --- |
| - histogram\_plot() (PlottingFunctions.Plotter method) |

## I

|  |  |
| --- | --- |
| - image\_plot() (PlottingFunctions.Plotter method) - image\_scatter\_plot() (PlottingFunctions.Plotter method) - infocus\_indices() (AnalysisFunctions.Analysis\_Functions method) | - intensity\_pixel\_indices() (AnalysisFunctions.Analysis\_Functions method) - IO\_Functions (class in IOFunctions) - IOFunctions   - module |

## L

|  |
| --- |
| - load\_json() (IOFunctions.IO\_Functions method) |

## M

|  |
| --- |
| - make\_datarray\_cell() (AnalysisFunctions.Analysis\_Functions method) - make\_datarray\_spot() (AnalysisFunctions.Analysis\_Functions method) - make\_directory() (IOFunctions.IO\_Functions method) - module   - AnalysisFunctions   - IOFunctions   - PlottingFunctions   - RASPRoutines |

## P

|  |  |
| --- | --- |
| - Plotter (class in PlottingFunctions) | - PlottingFunctions   - module |

## R

|  |  |
| --- | --- |
| - RASP\_Routines (class in RASPRoutines) - RASPRoutines   - module | - read\_tiff() (IOFunctions.IO\_Functions method) - read\_tiff\_tophotons() (IOFunctions.IO\_Functions method) - rejectoutliers() (AnalysisFunctions.Analysis\_Functions method) - ricker\_wavelet() (AnalysisFunctions.Analysis\_Functions method) |

## S

|  |  |
| --- | --- |
| - save\_analysis\_params() (IOFunctions.IO\_Functions method) - save\_analysis\_results() (RASPRoutines.RASP\_Routines method) - save\_analysis\_results\_onesavefile() (RASPRoutines.RASP\_Routines method) | - save\_as\_json() (IOFunctions.IO\_Functions method) - single\_image\_analysis() (RASPRoutines.RASP\_Routines method) - small\_feature\_kernel() (AnalysisFunctions.Analysis\_Functions method) |

## T

|  |  |
| --- | --- |
| - test\_spot\_mask\_overlap() (AnalysisFunctions.Analysis\_Functions method) | - two\_column\_plot() (PlottingFunctions.Plotter method) |

## W

|  |
| --- |
| - write\_tiff() (IOFunctions.IO\_Functions method) |

---

© Copyright 2024, Joseph S. Beckwith, Bin Fu, Steven F. Lee.

Built with Sphinx using a
theme
provided by Read the Docs.
