# Supplementary material for: RASP: Optimal Single Puncta Detection in Complex Cellular Backgrounds
Source: J Phys Chem B. 2024 Apr 9;128(15):3585–97. doi: 10.1021/acs.jpcb.4c00174 (PMC11033865; doi:10.1021/acs.jpcb.4c00174)
Supplement: Supplementary file 3 — jp4c00174_si_003.zip [file jp4c00174_si_003.zip › pyRASP_zip/docs/_build/html/getting-started.html]

Introduction — pyRASP v0.5.0 documentation


pyRASP

Contents:

- Introduction
  - Limitations
  - Getting Started with pyRASP
- src

pyRASP

- Introduction
- View page source

---

# Introduction

`pyRASP` is a Python package written in support of the “RASP” work from the lab of Steven F. Lee. The code is a set of python classes that can be run from scripts, interactive notebooks and so on to analyse microscopy data. An example notebook is provided, showing user analyses. The code has been tested in Python 3.10.12.

A list of package requirements are noted in the “requirements.txt” file. These can be installed with the command:

`pip install -r requirements.txt`

The current implementation has been developed in Python 3 and tested on an Ubuntu 20.04 machine, as well as several Windows 10 machines.

## Limitations

The RASP concept assumes that you are imaging something that is tissue/cell like, *i.e.* has autofluorescence. If you run your radiality calibration on data that do not contain autofluorescence, likely the code will pick out genuinely fluorecent spots (if you have any) and so remove real stuff from your data.

## Getting Started with pyRASP

Previous
Next

---

© Copyright 2024, Joseph S. Beckwith, Bin Fu, Steven F. Lee.

Built with Sphinx using a
theme
provided by Read the Docs.
