# Supplementary material for: RASP: Optimal Single Puncta Detection in Complex Cellular Backgrounds
Source: J Phys Chem B. 2024 Apr 9;128(15):3585–97. doi: 10.1021/acs.jpcb.4c00174 (PMC11033865; doi:10.1021/acs.jpcb.4c00174)
Supplement: Supplementary file 3 — jp4c00174_si_003.zip [file jp4c00174_si_003.zip › pyRASP_zip/docs/_build/html/index.html]

Welcome to pyRASP’s documentation! — pyRASP v0.5.0 documentation


pyRASP

Contents:

- Introduction
- src

pyRASP

- Welcome to pyRASP’s documentation!
- View page source

---

# Welcome to pyRASP’s documentation!

Contents:

- Introduction
  - Limitations
  - Getting Started with pyRASP
- src
  - AnalysisFunctions module
  - IOFunctions module
  - PlottingFunctions module
  - RASPRoutines module

# Indices and tables

- Index
- Module Index
- Search Page

Next

---

© Copyright 2024, Joseph S. Beckwith, Bin Fu, Steven F. Lee.

Built with Sphinx using a
theme
provided by Read the Docs.
