# Supplementary material for: RASP: Optimal Single Puncta Detection in Complex Cellular Backgrounds
Source: J Phys Chem B. 2024 Apr 9;128(15):3585–97. doi: 10.1021/acs.jpcb.4c00174 (PMC11033865; doi:10.1021/acs.jpcb.4c00174)
Supplement: Supplementary file 3 — jp4c00174_si_003.zip [file jp4c00174_si_003.zip › pyRASP_zip/docs/_build/html/modules.html]

src — pyRASP v0.5.0 documentation


pyRASP

Contents:

- Introduction
- src
  - AnalysisFunctions module
  - IOFunctions module
  - PlottingFunctions module
  - RASPRoutines module

pyRASP

- src
- View page source

---

# src

- AnalysisFunctions module
  - `Analysis_Functions`
    - `Analysis_Functions.Gauss2DFitting()`
    - `Analysis_Functions.bincalculator()`
    - `Analysis_Functions.calculate_gradient_field()`
    - `Analysis_Functions.calculate_mask_fill()`
    - `Analysis_Functions.calculate_radiality()`
    - `Analysis_Functions.calculate_region_properties()`
    - `Analysis_Functions.calculate_spot_colocalisation_likelihood_ratio()`
    - `Analysis_Functions.compute_image_props()`
    - `Analysis_Functions.compute_spot_and_cell_props()`
    - `Analysis_Functions.compute_spot_props()`
    - `Analysis_Functions.create_filled_region()`
    - `Analysis_Functions.create_gaussian_kernel()`
    - `Analysis_Functions.create_kernel()`
    - `Analysis_Functions.default_spotanalysis_routine()`
    - `Analysis_Functions.detect_large_features()`
    - `Analysis_Functions.dilate_pixel()`
    - `Analysis_Functions.estimate_intensity()`
    - `Analysis_Functions.gen_CSRmats()`
    - `Analysis_Functions.generate_mask_and_spot_indices()`
    - `Analysis_Functions.infocus_indices()`
    - `Analysis_Functions.intensity_pixel_indices()`
    - `Analysis_Functions.make_datarray_cell()`
    - `Analysis_Functions.make_datarray_spot()`
    - `Analysis_Functions.rejectoutliers()`
    - `Analysis_Functions.ricker_wavelet()`
    - `Analysis_Functions.small_feature_kernel()`
    - `Analysis_Functions.test_spot_mask_overlap()`
- IOFunctions module
  - `IO_Functions`
    - `IO_Functions.load_json()`
    - `IO_Functions.make_directory()`
    - `IO_Functions.read_tiff()`
    - `IO_Functions.read_tiff_tophotons()`
    - `IO_Functions.save_analysis_params()`
    - `IO_Functions.save_as_json()`
    - `IO_Functions.write_tiff()`
- PlottingFunctions module
  - `Plotter`
    - `Plotter.histogram_plot()`
    - `Plotter.image_plot()`
    - `Plotter.image_scatter_plot()`
    - `Plotter.two_column_plot()`
- RASPRoutines module
  - `RASP_Routines`
    - `RASP_Routines.analyse_images()`
    - `RASP_Routines.analyse_round_images()`
    - `RASP_Routines.analyse_round_subfolder()`
    - `RASP_Routines.calibrate_area()`
    - `RASP_Routines.calibrate_radiality()`
    - `RASP_Routines.count_spots()`
    - `RASP_Routines.file_search()`
    - `RASP_Routines.get_infocus_planes()`
    - `RASP_Routines.save_analysis_results()`
    - `RASP_Routines.save_analysis_results_onesavefile()`
    - `RASP_Routines.single_image_analysis()`

Previous
Next

---

© Copyright 2024, Joseph S. Beckwith, Bin Fu, Steven F. Lee.

Built with Sphinx using a
theme
provided by Read the Docs.
