# Supplementary material for: RASP: Optimal Single Puncta Detection in Complex Cellular Backgrounds
Source: J Phys Chem B. 2024 Apr 9;128(15):3585–97. doi: 10.1021/acs.jpcb.4c00174 (PMC11033865; doi:10.1021/acs.jpcb.4c00174)
Supplement: Supplementary file 3 — jp4c00174_si_003.zip [file jp4c00174_si_003.zip › pyRASP_zip/docs/_build/html/py-modindex.html]

Python Module Index — pyRASP v0.5.0 documentation


pyRASP

Contents:

- Introduction
- src

pyRASP

- Python Module Index

---

# Python Module Index

**a** |
**i** |
**p** |
**r**

|  |  |  |
| --- | --- | --- |
|  |  |  |
|  | **a** |  |
|  | `AnalysisFunctions` |  |
|  |  |  |
|  | **i** |  |
|  | `IOFunctions` |  |
|  |  |  |
|  | **p** |  |
|  | `PlottingFunctions` |  |
|  |  |  |
|  | **r** |  |
|  | `RASPRoutines` |  |

---

© Copyright 2024, Joseph S. Beckwith, Bin Fu, Steven F. Lee.

Built with Sphinx using a
theme
provided by Read the Docs.
