# Supplementary material for: RASP: Optimal Single Puncta Detection in Complex Cellular Backgrounds
Source: J Phys Chem B. 2024 Apr 9;128(15):3585–97. doi: 10.1021/acs.jpcb.4c00174 (PMC11033865; doi:10.1021/acs.jpcb.4c00174)
Supplement: Supplementary file 3 — jp4c00174_si_003.zip [file jp4c00174_si_003.zip › pyRASP_zip/docs/_build/html/search.html]

Search — pyRASP v0.5.0 documentation


pyRASP

Contents:

- Introduction
- src

pyRASP

- Search

---

Please activate JavaScript to enable the search functionality.

---

© Copyright 2024, Joseph S. Beckwith, Bin Fu, Steven F. Lee.

Built with Sphinx using a
theme
provided by Read the Docs.
